# Synthesis of Highly Oxygenated Bicyclic Carbasugars. Remarkable Difference in the Reactivity of the d-*gluco* and d*-xylo-* Derived Trienes

**DOI:** 10.3390/molecules25153357

**Published:** 2020-07-24

**Authors:** Grzegorz Witkowski, Mykhaylo A. Potopnyk, Karolina Tiara, Anna Osuch-Kwiatkowska, Sławomir Jarosz

**Affiliations:** Institute of Organic Chemistry, Polish Academy of Sciences, Kasprzaka 44/52, 01-224 Warsaw, Poland; grzegorz.witkowski@icho.edu.pl (G.W.); mpotopnyk@icho.edu.pl (M.A.P.); karolina.tiara@icho.edu.pl (K.T.); aosuch@icho.edu.pl (A.O.-K.)

**Keywords:** bicyclic sugar mimetics, stereoselective synthesis, Diels−Alder reaction, Wittig-type reaction

## Abstract

2,3,4-Tri-*O*-benzyl-D-xylopyranose was used as a starting material in the preparation of the corresponding triene, which underwent smooth cyclization to a polyhydroxylated hydrindane, as a single diastereoisomer. The analogous triene prepared from D-glucose did not undergo any cyclization even under high pressure.

## 1. Introduction

**Scheme 1:**
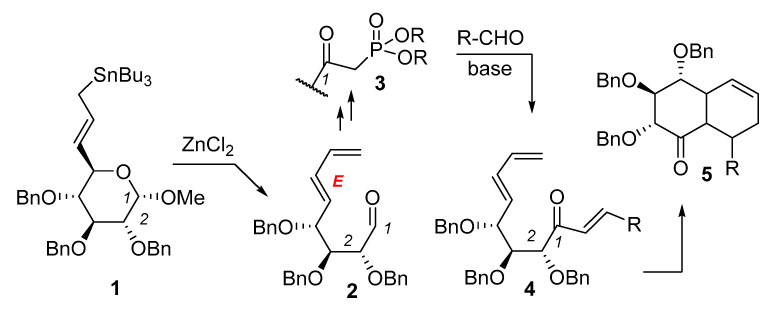
Synthesis of polyhydroxylated decalins from sugar allyltins.

Carbasugars are glycomimetics obtained through a substitution of the endocyclic oxygen atom with a methylene group [1,2]. Due to their similarity to carbohydrates, these compounds often possess interesting biological properties, e.g., they may act as glycosidase inhibitors [3,4,5]. Some of these compounds found an application in medicine; for example, acarbose is commercialized to treat obesity and type 2 diabetes mellitus [6] and validamycin is an antibiotic [7]. Elaboration of the convenient synthetic methods for the preparation of carbobicyclic sugar mimetics is one of the leading trends in our laboratory [8]. We have proposed a useful route to polyhydroxylated decalins (such as **5**) from sugar allyltins as shown in Scheme 1 [9]. The model synthesis was initiated from D-*gluco*-derivative **1**, which—upon treatment with mild Lewis acid (preferably ZnCl_2_)—afforded dienoaldehyde **2** with the *E*-configuration across the internal double bond. This compound was converted into phosphonate **3** and—by the Horner-Wadsworth-Emmons (HWE) reaction with the corresponding aldehyde—into triene **4**, subsequently cyclized to **5**. The methodology proposed in Scheme 1 allowed the preparation of a number of diastereoisomeric polyhydroxylated bicyclic derivatives in optically pure form starting from different sugars: d-glucose, d-mannose, and d-galactose [9,10,11,12].

It is known that polyhydroxylated decalins might have significant biological activity [13,14,15], thus there is an interest in the preparation of such targets. Our methodology, however, starting from organostannanes—being assumed to be highly toxic—cannot be used for the preparation of potential therapeutics. Other, environmentally friendly method(s), for the synthesis of such important derivatives is needed.

**Scheme 2:**
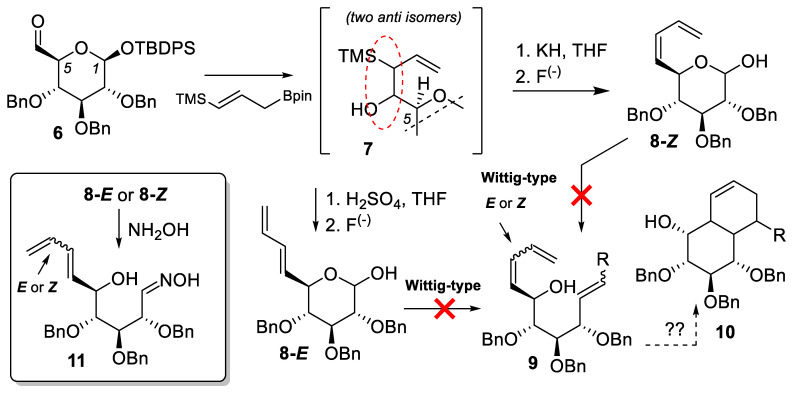
Stereoselective synthesis of sugar dienes **8**-***E*/8**-***Z*** and their reactivity.

We have proposed an efficient method for the stereoselective preparation of sugar dienes with the *E* or *Z* geometry across the internal double bond (**8**-***E*** or **8**-***Z***; Scheme 2) from D-glucosep-derivative **6** (via intermediate **7**) [16]. These compounds could be eventually used as convenient precursors for the synthesis of bicyclic carbasugars. Wittig-type reaction of hemiacetal **8** could provide appropriate triene **9** which, via a [4 + 2]-cyclization, should afford carbobicyclic derivatives (e.g., **10**; Scheme 2).

However, treatment of compounds **8** (either ***E*** or ***Z***) with phosphoranes (Wittig reaction) or phosphonates (HWE reaction) did not give any expected product **9**. Further, these hemiacetals did not react even with simplest organometallic species such e.g., Grignard reagents. The only process which could be realized, was the reaction with hydroxylamine providing the corresponding oxime **11** in good yield (Scheme 2).

## 2. Results and Discussion

**Scheme 3:**
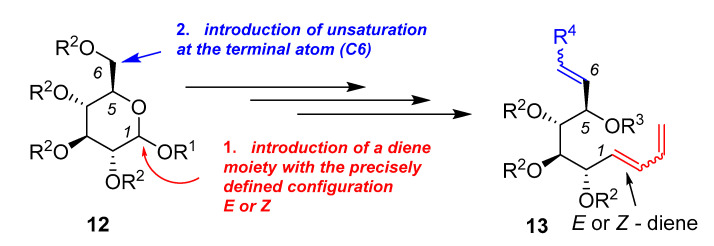
A concept of the synthesis of ‘inverted’ sugar trienes from d-glucose.

Since the intermediate **9** (see Scheme 2) could not be obtained from our diene **8**, we changed the concept and design to introduce the diene system at the anomeric position and then the olefinic unit at the terminal position; this triene can be called “inverted” comparing to triene **9**. This strategy is exemplified on Scheme 3 for D-glucose derivative **12**. We reasoned that this approach would be more profitable, the problems encountered for diene **8** could be avoided, and we would be able to obtain triene **13** in reasonable yield.

**Scheme 4:**
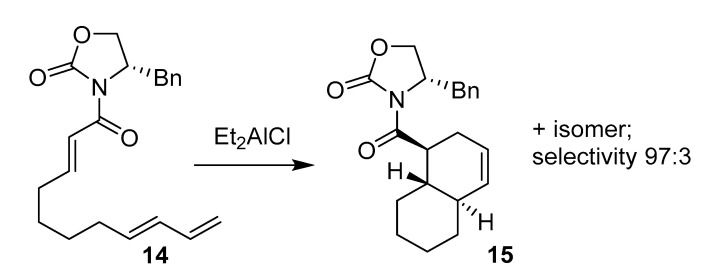
Cyclization of the triene **14** by Evans et. al. ref. [17,18,19].

This triene should also cyclize to the bicyclic system in the Diels-Alder reaction. This assumption is based on the results of Evans, who presented an efficient and highly diastereoselective cyclization of similar triene with the activating *N*-acyloxazolidinone unit at the olefinic part (**14**→**15**; Scheme 4) [17,18,19].

### 2.1. Synthesis of Derivatives of D-Glucose with the Diene System Located at the C1

**Scheme 5:**
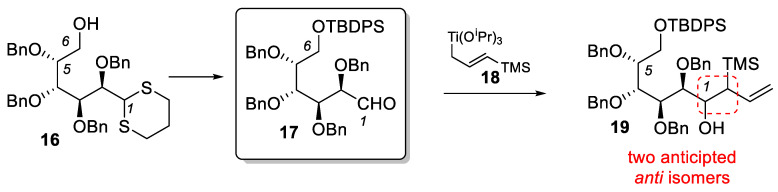
Synthesis of silanol **19**.

The synthesis of polyhydroxylated trienes in our case was initiated from the easily available di-thioacetal **16**, prepared from free glucose according to Yu and coworkers [20]. The OH group at the terminal position in **16** was protected as a TBDPS ether, the carbonyl group at the C1 was regenerated according to reference [21], and the resulting aldehyde **17** was reacted with allyltitanium reagent **18 [22]**, providing the mixture of silanols **19** with the anticipated [16,22,23] anti-configuration (Scheme 5). 

**Scheme 6:**
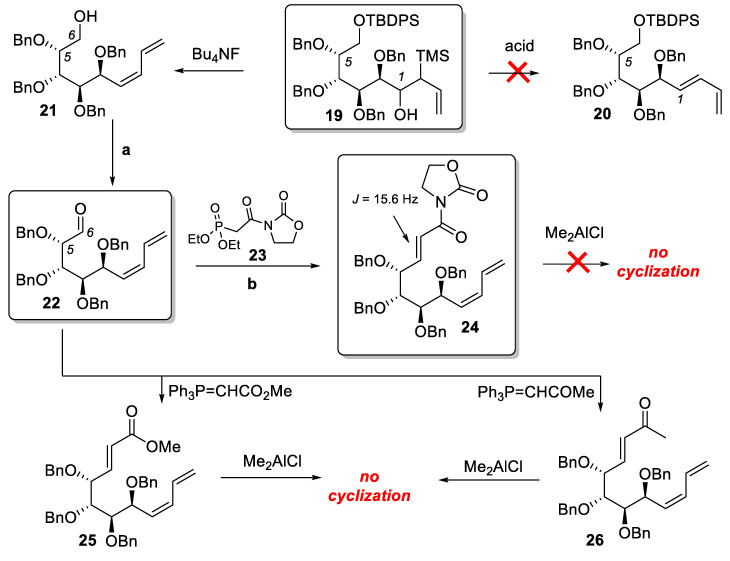
(**a**) TEMPO (2,2,6,6-tetrametylopiperydyno-1-oksyl), TCICA (trichloroisocyanuric acid), 0 °C to rt., 15 min.; (**b**) **23**, LiBr, i-Pr_2_NEt, MeCN, rt., 18 h.

β-Hydroxysilane 19 was subjected to the Peterson olefination under acidic and, independently, basic conditions which should lead to the corresponding dienes with the *E* or *Z*-configuration across the internal double bond. However, treatment of 19 with acids (H_2_SO_4_ or BF_3_) did not provide diene 20; the starting material remained unchanged (Scheme 6).

We reasoned that this resistance to the elimination of silanol might result from the presence of the bulky substituent located at the terminal position (TBDPS ether), so deprotection of this position might solve the problem. Treatment of **19** with fluoride anion not only removed this silyl group but also induced the Peterson olefination providing the *Z*-diene **21**. This *Z*-diene was thus used to test our hypothesis (shown in Scheme 3) of the convenient preparation of the “inverted” trienes and their subsequent application in the synthesis of bicyclic derivatives via a cyclization reaction as reported by Evans for triene **14** (cf. Scheme 4).

Alcohol **21** was oxidized to the corresponding aldehyde **22** which reacted with phosphonate **23** [24], to afford triene **24** with the *E*-configuration across the newly created double bond (Scheme 6). This triene was subjected to the cyclization induced by Me_2_AlCl; no reaction was, however, noticed. Application of other Lewis acids, as well as, high pressure (10 kbar) also did not give any positive result. This lack of cyclization was very surprising, especially in the context of the results obtained by Evans.

We prepared also two other trienes: **25** and **26** by reaction of aldehyde **22** with appropriate phosphoranes: Ph_3_P=CHCO_2_Me and Ph_3_P=CHCO_2_Me. None of the resulting trienes, however, underwent cyclization (Scheme 6).

Why could the cyclization of compounds—similar to those successfully used in Evans’ synthesis—not be realized? Is it a result of only the different configuration across the double bond in a diene system (*Z* in **24** in our case vs. *E* in **14** in Evans case)? Or maybe other reasons are responsible for that strange result. This phenomenon is not clear.

We turned, therefore, our attention to shorter analogs being precursors of perhydroindane derivatives.

### 2.2. Synthesis of Derivatives of D-Xylose with the Diene System at the C1

**Scheme 7:**
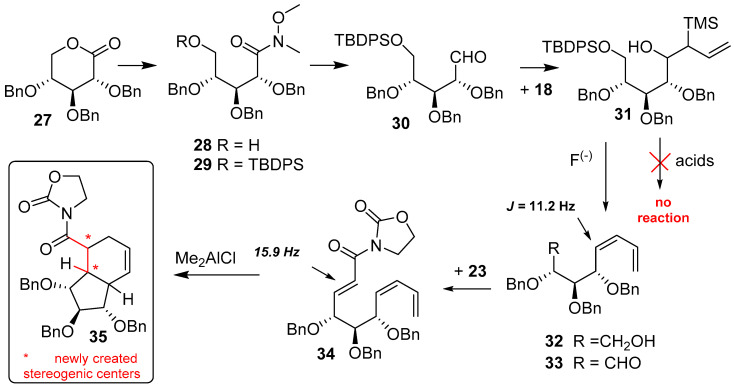
Stereoselective synthesis of bicyclic derivative **35** from d-xylose.

The synthesis was initiated from known lactone **27 [25]**, obtained by an oxidation of 2,3,4-tri-*O*-benzyl-D-xylose. It was reacted with the in situ generated complex of Me_3_Al and *N*,*O*-dimethylhydroxylamine which provided the Weinreb amide **28**. Since this compound was relatively unstable (easily underwent decomposition to **27**), it was converted immediately into silylated derivative **29** and then into aldehyde **30**. Reactivity of **30** was similar to the previously obtained derivatives. Its conversion into **31** followed the same route as for D-glucose derivatives (depicted in Scheme 1); this silanol (**31**) was also unreactive under the acidic conditions and underwent conversion into *Z*-diene **32** with the simultaneous removal of the silyl block upon treatment with fluoride anion (Scheme 7). The configuration of the internal double bond in the diene part was proven by ^1^H NMR data in which the coupling constant *J* = 11.2 Hz was observed (see Scheme 7).

Compound **32** was converted into aldehyde **33** and further into triene **34**, with the *E*-configuration across the newly created double bond (*J* = 15.9 Hz). Cyclization of this triene induced with Me_2_AlCl provided bicyclic compound **35** as a single stereoisomer (Scheme 3); only two olefinic signals at δ = 128.0 and 124.3 ppm were seen in the ^13^C NMR spectrum.

**Figure 1:**
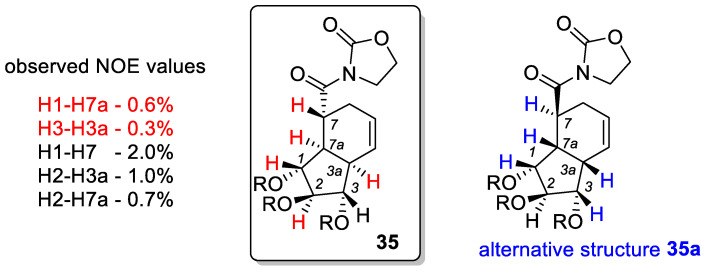
The NOE values of bicyclic product **35.**

The configuration of the product was assigned by the Nuclear Overhauser Effect (NOE) measurement (Figure 1).

Small NOE values between the H1-H7a and H3-H3a confirmed structure **35** and—at the same time—excluded the alternative one: **35a** in which the strong H1-H7a and H3-H3a interaction should have been observed.

## 3. Materials and Methods

### 3.1. General

NMR spectra were recorded in CDCl_3_ (internal Me_4_Si) with a Varian AM-600 (600 MHz ^1^H, 150 MHz ^13^C) spectrometer (Sugar Land, TX, USA) at rt. Chemical shifts (δ) are reported in ppm relative to Me_4_Si (δ 0.00) for ^1^H and residual chloroform (δ 77.00) for ^13^C. All significant resonances (carbon skeleton) were assigned by COSY (^1^H-^1^H), HSQC (^1^H-^13^C), and HMBC (^1^H-^13^C) correlations. The relative configuration of the stereogenic centers was assigned on the basis of 1D-NOESY spectra. Mass spectra (ESI) were recorded with an Applied Biosystems 4000 Q-TRAP (low resolution) (Toronto, ON, Canada) and Waters AutoSpec Premier (Waters, Milford, MA, USA) or Waters MALDISynapt G2-S HDMS (high resolution) spectrometers (Manchester, UK). HPLC analyses were conducted on Merck-Hitachi apparatus (Darmstadt, Germany) equipped with Merck LiChrospher 100 RP-18 (250 × 0.4 mm, 5 μm) column and UV detector. Column chromatography was performed on silica gel 60 (70–230 mesh, Merck. Flash chromatography was performed on Buchi glass columns packed with silica gel 60 (230–400 mesh, Merck), using Knauer Smartline system with a Buchi fraction collector. Reagents were purchased from Sigma-Aldrich (St. Louis, MO, USA), Alfa Aesar (Tianjin, China) or ABCR (Karlsruhe, Germany), and used without purification. Thin-layer chromatography was carried out on silica gel 60 F_254_ (Merck). The organic solutions were dried over MgSO_4_.

The scans of the NMR data for all compounds are provided as Supplementary Materials.

#### 3.1.1. 2,3,4,5-Tetra-*O*-benzyl-6-*O*-*tert*-butyl-diphenylsilyl-D-glucose **17**

To a cooled to 0 °C solution of 2,3,4,5-tetra-*O*-benzyl-D-glucose propane-1,3-diyl dithioacetal [20] (**16**; 21.89 g; 34.7 mmol) and imidazole (4.74 g, 69.4 g, 2.0 eq) in methylene chloride (300 mL), *tert*-butyl-diphenylsilyl chloride (9.6 mL, 37.5 mmol, 1.1 equiv.) was added dropwise within 1 h and the mixture was stirred overnight at room temperature. Water (150 mL) was added, the layers were separated and the organic solution was washed with 1M H_2_SO_4_ (100 mL) and water (100 mL), dried, concentrated, and the crude product was isolated by column chromatography (hexanes-ethyl acetate 7:1→3:1) to afford the 6-*O*-protected derivative (27.47 g; 91%) as an oil. 

^1^H NMR δ: 7.69−7.52 (m, 4H), 7.48−7.15 (m, 26H), 4.88 (d, *J* = 11.5 Hz, 1H), 4.76−4.57 (m, 5H), 4.49 (d, *J* = 11.2 Hz, 1H), 4.38 (d, *J* = 11.5 Hz, 1H), 4.23 (d, *J* = 4.8 Hz, 1H), 4.07 (t, *J* = 5.2 Hz, 1H), 3.99−3.91 (m, 2H), 3.85−3.81 (m, 1H), 3.69 (dd, *J* = 6.2, 4.6 Hz, 1H), 3.54 (dt, *J* = 5.6, 4.3 Hz, 1H), 2.87−2.74 (m, 1H), 2.77−2.53 (m, 3H), 1.97−1.91 (m, 1H), 1.91−1.81 (m, 1H), 1.10 (s, 9H);

^13^C NMR δ: 138.9, 138.7, 2 × 138.4, 135.9–127.0, 81.9, 79.8, 79.9, 78.4, 75.2, 74.7, 73.8, 72.5, 62.4, 49.5, 30.0, 29.5, 27.0, 26.2, 19.3.

HR-MS: *m/z* = 868.3647; calcd for C_53_H_60_O_5_S_2_SiNa [M + Na]^+^: 868.3651. 

**Figure 2:**
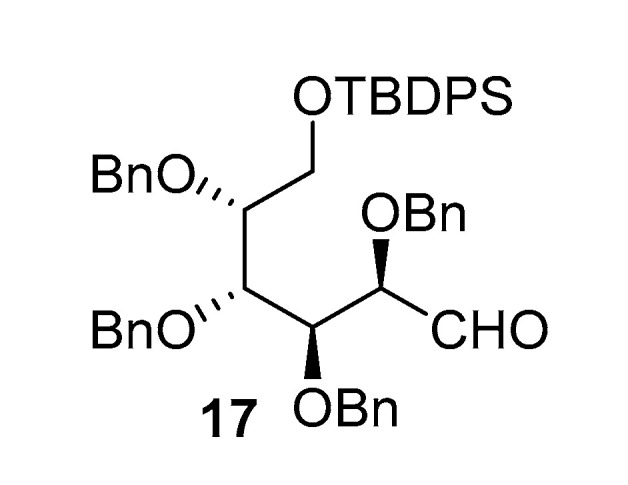
The structure of aldehyde **17**.

To a solution of the above prepared compound (4.36 g; 5.02 mmol) in MeCN:CH_2_Cl_2_:H_2_O (8:1:1; 50 mL), Dess-Martin reagent (4.47 g, 10.0 mmol, 2.0 eq) was added, the mixture was stirred at room temperature overnight, and concentrated. The residue was purified by column chromatography (hexanes-ethyl acetate 7:1→3:1) affording the title product **17** (3.28 g; 84%) (Figure 2), which was used immediately in the next step.

LR-MS: *m/z* = 833 [C_51_H_58_O_7_SiNa (M + MeOH+Na)^+^].

#### 3.1.2. Olefin **19**

Generation of the titanium reagent **18**: To a cooled to −78 °C solution of allyltrimethylsilane (3.7 g, 32.4 mmol, 10.0 eq) in dry THF (32 mL), a solution of 2.5 M BuLi in hexane (12 mL, 29.1 mmol, 9.0 eq.) was added within 1h with a syringe pump; after another 30 min. the mixture became yellowish. Then, a solution of 1.0 M ClTi(O*^i^*Pr)_3_ in methylene chloride (29 mL) was added within 30 min (syringe pump) and the red mixture was stirred for another 30 min.

**Figure 3:**
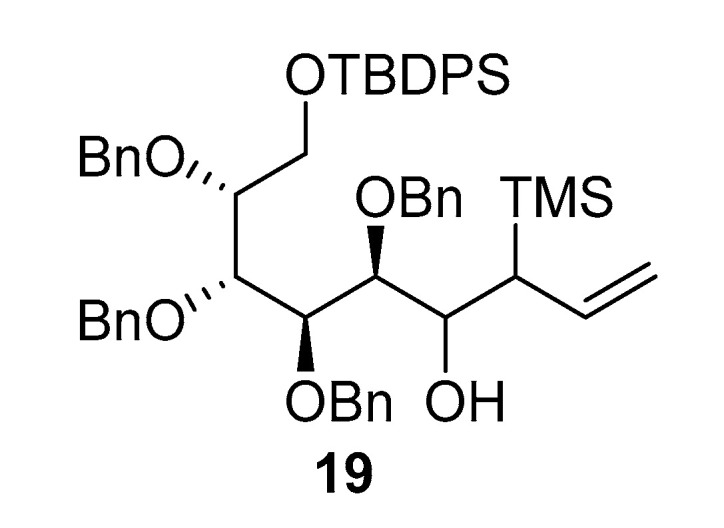
The structure of silanol **19**.

To such prepared reagent **18**, a solution of **17** (2.10 g, 3.19 mmol) in dry THF (6.4 mL) was added within 1h, the mixture was stirred overnight at −78 °C (using immersion cooler with temperature control Huber TC100E, temperature range −100 to +40 °C) and partitioned between water (70 mL) and ether (70 mL). The white precipitate was filtered off and discarded; the organic layer was separated, washed with brine, and concentrated to afford a crude mixture of (anticipated) anti-isomers, pure enough to be used in the next steps (Figure 3).

LR-MS: *m/z* = 915 [C_56_H_68_O_6_Si_2_Na (M + Na)^+^].

#### 3.1.3. Dienoalcohol **21**

**Figure 4:**
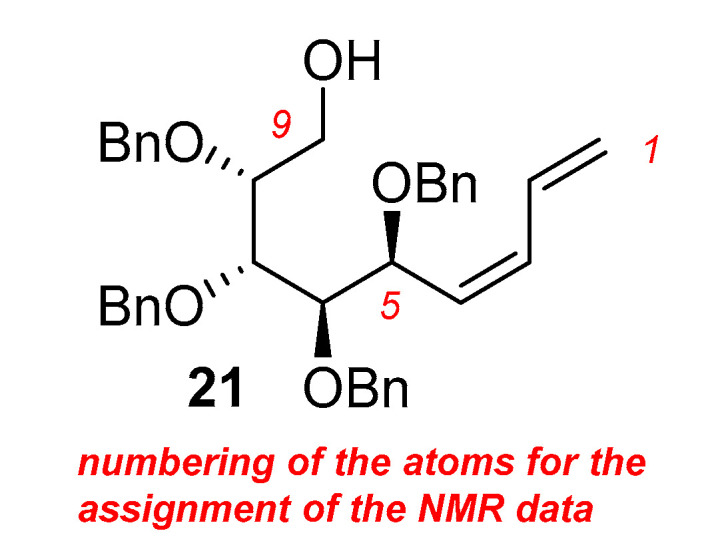
The structure of diene **21** and the numbering of the atoms.

To a solution of crude **19** obtained above in THF (16 mL) a solution of 1.0 M TBAF in THF (16 mL, 16.0 mmol, 5.0 eq.) was added and the mixture was stirred at room temperature overnight. Then it was concentrated and the residue was subjected to column chromatography (hexane-ethyl acetate, 92:8→36:64) to afford the title product **21** (1.11 g, 73% over two steps) as an oil (Figure 4).

^1^H NMR δ: 7.39–7.17 (m, 20H, arom.), 6.51 (ddd, *J* = 16.6, 11.0 and 10.1 Hz, 1H, H-2), 6.29 (t, *J* = 11.2 Hz, 1H, H-3), 5.46 (t, *J* = 10.3 Hz, 1H, H-4), 5.28 (d, *J* = 16.7 Hz, 1H, H-1), 5.11 (d, *J* = 10.1 Hz, 1H, H-1′), 4.86 (d, *J* = 11.4 Hz, 1H, OC*H*_2_Ph), 4.73 (d, *J* = 11.4 Hz, 1H, OC*H*_2_Ph), 4.68 (d, *J* = 11.5 Hz, 1H, OC*H*_2_Ph), 4.63–4.57 (m, 3H, H-5 and 2×OC*H*_2_Ph), 4.49 (d, *J* = 11.6 Hz, 1H, OC*H*_2_Ph), 4.37 (d, *J* = 11.6 Hz, 1H, OC*H*_2_Ph), 4.33 (d, *J* = 11.7 Hz, 1H, OC*H*_2_Ph), 3.90 (t, *J* = 4.9 Hz, 1H, H-8), 3.85 (ddd, *J* = 12.0, 5.4, and 4.0 Hz, 1H, H-9), 3.77 (ddd, *J* = 11.7, 6.9, 4.0 Hz, 1H, H-9′), 3.73 (dd, *J* = 6.1, 4.4 Hz, 1H, H-6), 3.66 (dt, *J* = 5.5 and 4.0 Hz, 1H, H-7), 2.15 (dd, *J* = 6.9 and 5.5 Hz, 1H, OH); 

^13^C NMR δ: 138.8, 138.7 and 2 × 138.3(quat. benzyl), 134.2 (C-3), 131.9 (C-2), 128.6−127.6 (arom. and C-4), 120.4 (C-1), 81.9 (C-6), 79.7 (C-7), 79.3 (C-8), 75.6 (C-5), 75.2, 74.5, 71.7 and 70.5 (4×O*C*H_2_Ph), 60.8 (C-9).

HR-MS: *m/z* = 587.2770; calcd for C_37_H_40_O_5_Na [M + Na]^+^: 587.2773.

#### 3.1.4. Dienoaldehyde 22

**Figure 5:**
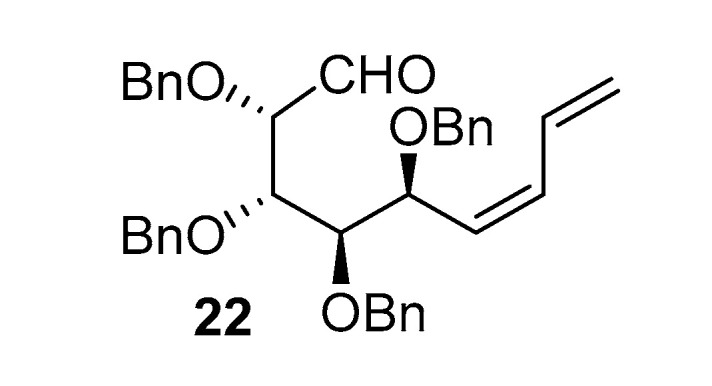
The structure of diene **19**.

To a cooled to 0 °C solution of alcohol **21** (350 mg, 0.62 mmol) and TEMPO (1.0 mg; 6.0 μmol) in dry CH_2_Cl_2_ (8 mL), trichloroisocyanuric acid (155 mg; 0.66 mmol; 1.1 eq) was added and the mixture was stirred for 15 min. Then it was filtered through short pad of Celite, the filtrate was diluted with Et_2_O (6.0 mL) and washed with 5% Na_2_S_2_O_3_ (2.0 mL), 1M NaOH (5 mL), 1M H_2_SO_4_ (5 mL), and water (3.0 mL). The organic phase was dried and concentrated, and the crude aldehyde was used immediately in the next step (Figure 5).

LR-MS: *m/z* = 617 [C_38_H_42_NO_6_Na (M + MeOH + Na)^+^].

#### 3.1.5. Triene **24**

**Figure 6:**
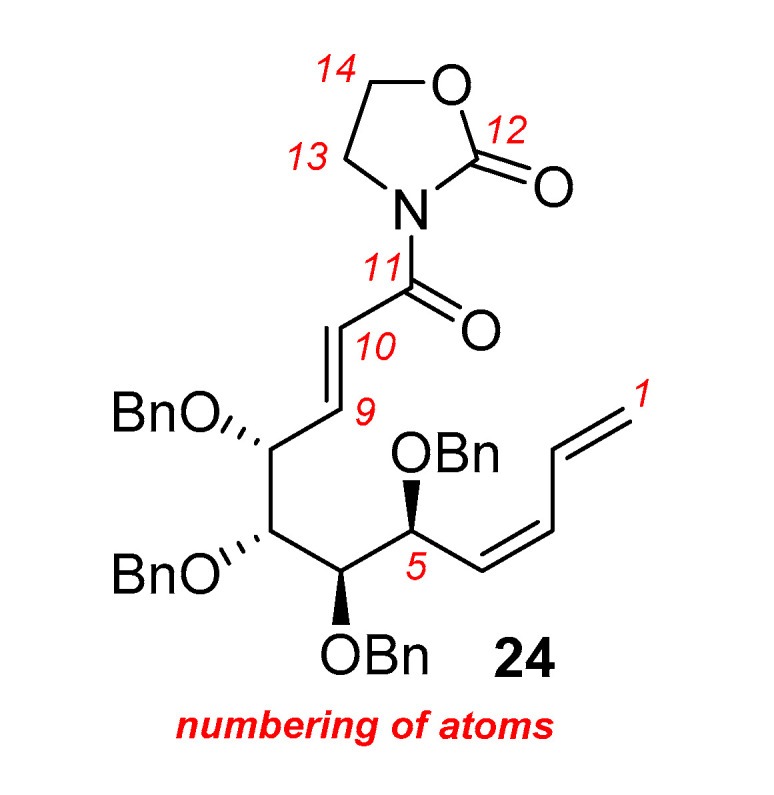
The structure of triene **24** and the numbering of the atoms.

To a solution of aldehyde **22** (170 mg, 0.3 mmol) and phosphonate **23** [21] (120 mg, 1.5 eq.) in dry CH_3_CN (3 mL), LiBr (40.5 mg, 0.466 mmol, 1.5 eq) was added followed by di-isopropylethylamine (80 μL, 0.466 mmol, 1.5 eq) and the mixture was stirred for 20 h. It was then concentrated and the crude product was purified by column chromatography (hexane 100% then: hexane-ethyl acetate 92:8→36:64) (Figure 6).

^1^H NMR δ: 7.46 (d, *J* = 15.6, 1H, H-10), 7.35–7.16 (m, 21H, arom. and H-9), 6.51 (ddd, *J* = 16.7, 11.3 and 10.1 Hz, 1H, H-2), 6.27 (t, *J* = 11.2 Hz, 1H, H-3), 5.45 (dd, *J* = 11.1 and 9.7 Hz, 1H, H-4), 5.25 (d, *J* = 16.7 Hz, 1H, H-1), 5.09 (d, *J* = 10.1 Hz, 1H, H-1′), 4.84 (d, *J* = 11.5 Hz, 1H, OC*H*_2_Ph), 4.67–4.59 (m, 3H, 2×OC*H*_2_Ph and H-5), 4.56 (d, *J* = 11.7 Hz, 1H, OC*H*_2_Ph), 4.55–4.47 (m, 2H, 2×OC*H*_2_Ph), 4.38–4.30 (m, 3H, OC*H*_2_Ph and 2×H-13), 4.24 (dd, *J* = 6.8 and 5.6 Hz, 1H, H-8), 4.14 (d, *J* = 11.4 Hz, 1H, OC*H*_2_Ph), 3.97 (qdd, *J* = 11.1, 8.9 and 7.1 Hz, 2H, 2×H-14), 3.81 (dd, *J* = 5.6 and 4.2 Hz, 1H, H-7), 3.74 (dd, *J* = 6.3 and 4.2 Hz, 1H, H-6); 

^13^C NMR δ: 164.5 (C-12), 153.3 (C-11), 147.6 (C-9), 138.9, 138.7, 138.4 and 137.9 (quat. benzyl), 134.1 (C-3), 132.1 (C-2), 128.6−127.3 (arom. and C-4), 122.3 (C-10), 120.2 (C-1), 82.0 (C-7), 81.6 (C-6), 79.2 (C-8), 75.5 (C-5), 75.3, 74.2, 71.4, 70.4 (4×O*C*H_2_Ph), 62.1 (C-13), 42.7 (C-14).

HR-MS: *m/z* = 696.2922; calcd for C_42_H_43_NO_7_Na [M + Na]^+^: 696.2937.

#### 3.1.6. Triene **25**

**Figure 7:**
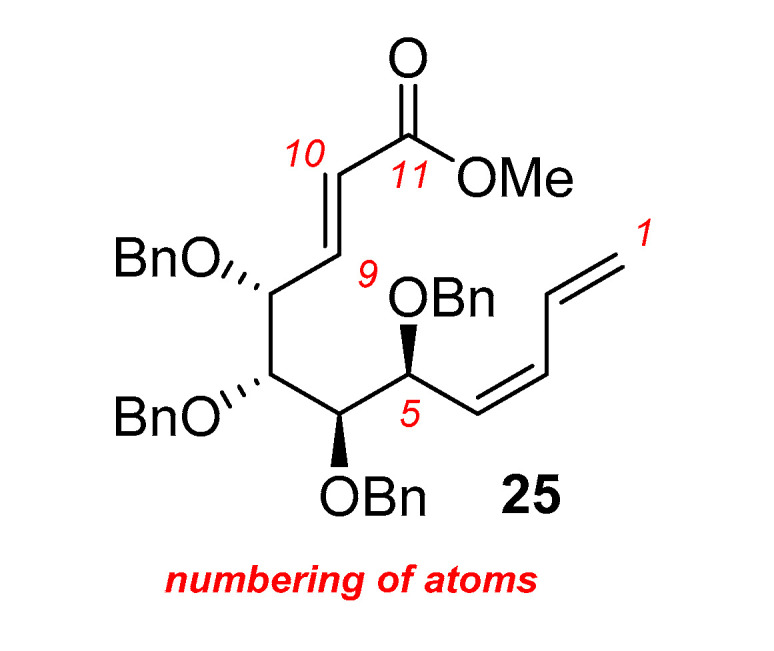
The structure of triene **25** and the numbering of the atoms.

Aldehyde **22** (45 mg) and Ph_3_P=CHCO_2_Me (54 mg; 2.0 eq.) were dissolved in dry benzene and stirred at room temperature for 16 h. The mixture was concentrated and the product was isolated by column chromatography (hexanes-ethyl acetate, 3:1→2:1) as a colorless oil in 74% yield (36.5 mg) (Figure 7).

^1^H NMR δ: 7.43–7.18 (m, 20H), 7.01 (dd, *J* = 15.8, 6.6 Hz, 1H, H-9), 6.45 (ddd, *J* = 16.7, 11.2, 10.0 and 6.0 Hz, 1H, H-2), 6.26 (t, *J* = 11.2 Hz, 1H, H-3), 6.01 (d, *J* = 15.9 Hz, 1H, H-10), 5.45 (t, *J* = 10.3 Hz, 1H, H-4), 5.26 (d, *J* = 16.7 Hz, 1H, H-1), 5.09 (d, *J* = 10.0 Hz, 1H, H-1′), 4.82 (d, *J* = 11.5 Hz, 1H, OC*H*_2_Ph), 4.63 (m, 2H, 2×OC*H*_2_Ph), 4.59–4.53 (m, 2H, OC*H*_2_Ph and H-5), 4.51 (d, *J* = 11.3 Hz, 1H, OC*H*_2_Ph), 4.46 (d, *J* = 11.5 Hz, 1H, OC*H*_2_Ph), 4.32 (d, *J* = 11.7 Hz, 1H, OC*H*_2_Ph), 4.15 (m, 2H, OC*H*_2_Ph and H-8), 3.81 (s, 3H, OMe), 3.79 (dd, *J* = 5.6 and 4.4 Hz, 1H, H-7), 3.73 (m, 1H, H-6);

^13^C NMR δ: 166.3 (C-11), 146.0 (C-9), 138.7, 138.4, 138.1 and 137.8 (quat. benzyl), 133.9 (C-3), 131.8 (C-2), 128.5−127.3 (arom. and C-4), 123.3 (C-10), 120.2 (C-1), 81.6 and 81.5 (C-6 and C-7), 78.8 (C-8), 75.2 (O*C*H_2_Ph), 75.1 (C-5), 74.1, 71.1,and 70.3 (3×O*C*H_2_Ph), 51.7 (OMe).

HR-MS: *m/z* = 641.2886; calcd for C_40_H_42_O_6_Na [M + Na]^+^: 641.2879.

#### 3.1.7. Triene **26**

**Figure 8:**
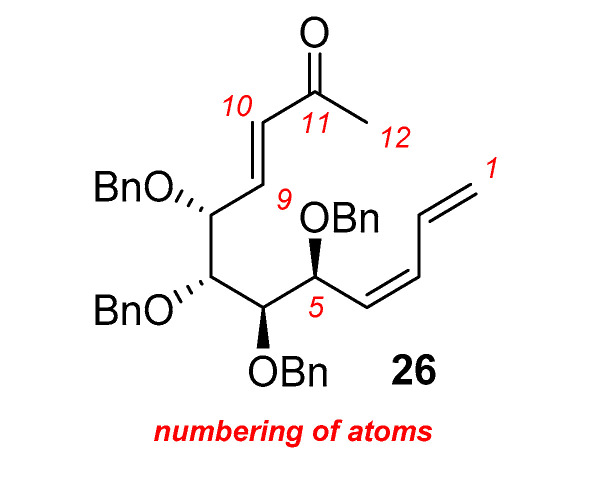
The structure of triene **26** and the numbering of the atoms.

This triene was prepared analogously as **25** from aldehyde **22** (47 mg) and Ph_3_P=CHCOMe (55 mg; 2.0 eq) as a colorless oil in 76% yield (36.5 mg) (Figure 8).

^1^H NMR δ: 7.36–7.19 (m, 20H, arom.), 6.78 (dd, *J* = 16.3, 6.5 Hz, 1H, H-9), 6.46 (dt, *J* = 16.2, 10.6 Hz, 1H, H-2), 6.28 (t, *J* = 11.1 Hz, 1H, H-3), 6.18 (d, *J* = 16.2 Hz, H-10), 5.45 (t, *J* = 10.4 Hz, 1H, H-4), 5.27 (d, *J* = 16.7 Hz, 1H, H-1), 5.11 (dd, *J* = 10.0 and 1.7 Hz, 1H, H-1′), 4.85 (d, *J* = 11.4 Hz, 1H, OC*H*_2_Ph), 4.64–4.56 (m, 4H, 3×OC*H*_2_Ph and H-5), 4.53 (d, *J* = 11.4 Hz, 1H, OC*H*_2_Ph), 4.45 (d, *J* = 11.6 Hz, 1H, OC*H*_2_Ph), 4.32 (d, *J* = 11.7 Hz, 1H, OC*H*_2_Ph), 4.18 (d, *J* = 11.6 Hz, 1H, OC*H*_2_Ph), 4.15 (ddd, *J* = 6.5 and 5.3 Hz, 1H, H-8), 3.81 (t, *J* = 4.8 Hz, 1H, H-7), 3.70 (dd, *J* = 6.3 and 4.3 Hz, 1H, H-6), 2.11 (s, 3H, H-12);

^13^C NMR δ: 198.2 (C-11), 144.7 (C-9), 138.6, 138.3, 138.1 and 137.8 (quat. benzyl), 134.1 (C-3), 132.6 (C-10), 131.7 (C-2), 128.5−127.4 (arom. and C-4), 120.3 (C-1), 81.7 and 81.6 (C-6 and C-7), 79.3 (C-8), 2 × 75.3 (C-5 and O*C*H_2_Ph), 74.0, 71.3, 70.3 (3×O*C*H_2_Ph), 26.9 (C-12).

HR-MS: *m/z* = 625.2936; calcd for C_40_H_42_O_5_Na [M + Na]^+^: 625.2930.

#### 3.1.8. Weinreb Amide **29**

To a cooled to 0 °C suspension of MeNHOMe x HCl (5.85 g, 60.0 mmol, 3.0 eq) in dry CH_2_Cl_2_ (175 mL), a 2M solution of Me_3_Al in toluene (30 mL, 60 mmol, 2 eq.) was added dropwise during 30 min. by a syringe pump. The mixture was stirred for an additional 30 min., then lactone **27** (8.37 g, 20 mmol) in dry CH_2_Cl_2_ (25 mL) was added within 30 min. by a syringe pump, and the mixture was stirred at room temperature for 3 h. Aqueous H_2_SO_4_ (1M solution, 100 mL) was carefully added and the organic phase was separated, washed with water (100 mL), brine (100 mL), and dried.

**Figure 9:**
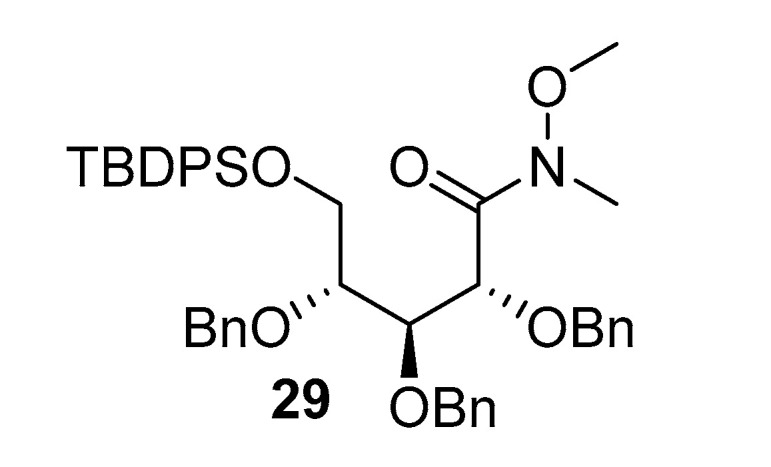
The structure of Weinreb amide **29**.

Imidazole (4.08 g, 60 mmol, 3.0 eq) was added to this containing crude **28** and the resulting mixture was cooled to 0 °C. A solution of t*ert*-butyldiphenylchlorosilane (7.8 mL, 30.0 mmol, 1.5 eq.) in CH_2_Cl_2_ (20 mL) was added dropwise within 1 h by a syringe pump and the mixture was stirred for additional 16 h. Aqueous H_2_SO_4_ (1M solution, 50 mL) was carefully added, the organic phase was separated, washed with water (100 mL), brine (100 mL), dried, and the crude product was purified by column chromatography (hexanes-ethyl acetate: 13:1→7:1) to give the title product **29** (12.35 g, 86% over two steps) as a colorless oil (Figure 9).

^1^H NMR δ: 7.49–7.42 (m, 3H, arom.), 7.36–7.22 (m, 15H, arom.), 7.18–7.15 (m, 2H, arom.), 4.78–4.71 (m, 2H, 2×OC*H*_2_Ph), 4.60–4.51 (m, 4H, H-5 and 3×OC*H*_2_Ph), 4.35 (d, *J* = 11.4 Hz, 1H, OC*H*_2_Ph), 3.72–3.65 (m, 3H, H-2, H-3 and H-4), 3.62 (s, 3H, OC*H*_3_), 3.41 (m, 1H, H-4′), 3.15 (bs, 3H, NC*H*_3_), 1.07 [s, 9H, OSiPh_2_C(C*H*_3_)_3_]; 

^13^C NMR δ 170.4 (C-1), 138.5, 138.3 and 138.0 (quat. benzyl), 135.8, 133.3, 129.6 and 129.3−126.5 (arom.), 82.3 (C-3), 79.6 (C-4), 76.1 (C-2), 74.2, 73.3 and 71.6 (3×O*C*H_2_Ph), 61.4 (O*C*H_3_), 32.8 (N*C*H_3_), 26.8 [OSiPh_2_C(*C*H_3_)_3_], 18.8 [OSiPh_2_*C*(CH_3_)_3_].

HR-MS: *m/z* = 740. 3387; calcd for C_44_H_51_NO_6_SiNa [M + Na]^+^: 740.3383.

#### 3.1.9. 2,3,4-Tri-*O*-benzyl-5-*O*-*tert*-butyl-diphenylsilyl-D-xylose **30**

**Figure 10:**
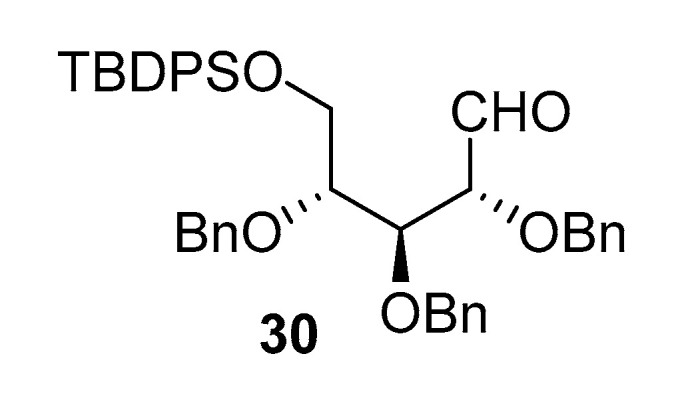
The structure of aldehyde **30.**

To a cooled to 0 °C solution of amide **29** (2.94 g, 4.10 mmol, 1.0 eq.) in dry Et_2_O (41 mL), LiAlH_4_ (0.19 g, 5.12 mmol, 1.25 eq.) was added in three portions within 15 min., and the mixture was stirred for 1 h. Aqueous H_2_SO_4_ (1M solution, 20 mL) was added, the organic phase was separated, washed with water (20 mL) and brine (20 mL), dried, and concentrated. The crude product was purified by column chromatography (hexanes→hexanes-ethyl acetate: 68:32) to give the aldehyde **30** (2.05 g, 76%) as a colorless oil (Figure 10). This product was used immediately in the next step.

LR-MS: *m/z =* 713 [C_43_H_50_O_6_SiNa (M + MeOH + Na)^+^].

#### 3.1.10. Silanol **31**

**Figure 11:**
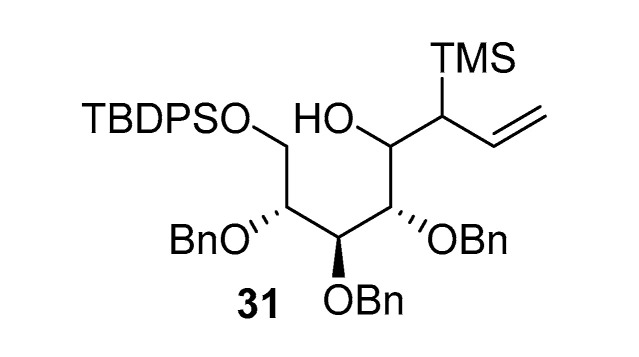
The structure of silanol **31.**

This compound was prepared analogously as **19**, from **30** (2.79 g, 4.23 mmol) as a colorless oil as a mixture of (anticipated) two anti-isomers (Figure 11).

LR-MS: *m/z* = 795 [C_48_H_60_O_5_Si_2_Na (M + Na)^+^].

#### 3.1.11. Dienoalcohol **32**

**Figure 12:**
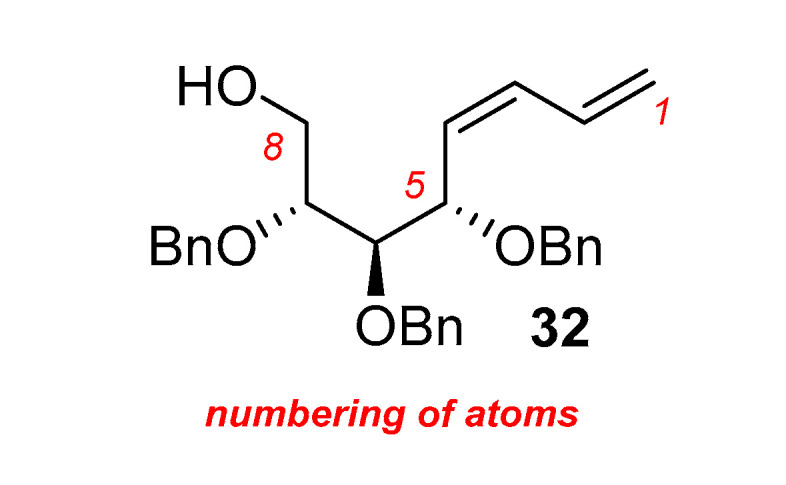
The structure of diene **32.**

This compound was prepared, analogously as **21**, from **31**. It was purified by column chromatography (hexanes→hexanes-ethyl acetate: 36:64) to give title dienalcohol **32** as a colorless oil (1.36 g, 72% over two steps) (Figure 12).

^1^H NMR δ: 7.37–7.22 (m, 15H, arom.), 6.58 (ddd, *J* = 16.7, 11.2 and 10.1 Hz, 1H, H-2), 6.27 (t, *J* = 11.2 Hz, 1H, H-3), 5.53 (t, *J* = 10.3 Hz, 1H, H-4), 5.29 (d, *J* = 16.7 Hz, 1H, H-1), 5.16 (d, *J* = 10.2 Hz, 1H, H-1′), 4.78 (d, *J* = 11.4 Hz, 1H, OC*H*_2_Ph), 4.73 (d, *J* = 11.4 Hz, 1H, OC*H*_2_Ph), 4.64–4.55 (m, 4H, H-5 and 3×OC*H*_2_Ph), 4.35 (d, *J* = 11.7 Hz, 1H, OC*H*_2_Ph), 3.72 (ddd, *J* = 11.3, 6.6 and 4.2 Hz, 1H, H-8), 3.69–3.65 (m, 2H, H-6 and H-7), 3.55 (ddd, *J* = 11.4, 5.6 and 3.5 Hz, 1H, H-8′), 2.12 (t, *J* = 6.3 Hz, 1H, OH); 

^13^C NMR δ: 138.6, 138.4 and 138.1 (quat. benzyl), 133.8 (C-3), 132.0 (C-2), 128.8−127.8 (arom. and C-4), 120.2 (C-1), 82.1 (C-6 or C-7), 79.7 (C-6 or C-7), 75.2 (O*C*H_2_Ph), 74.8 (C-5), 72.8 and 70.6 (2×O*C*H_2_Ph), 61.7 (C-8).

HR-MS: *m/z* = 467.2180; calcd for C_29_H_32_NO_4_Na [M + Na]^+^: 467.2188.

#### 3.1.12. Triene **34**

Alcohol **32** (244 mg, 0.55 mmol) was oxidized as described for **22** and the resulting product was purified by column chromatography (hexanes 100%→hexanes-ethyl acetate: 1:2) to afford the corresponding aldehyde **33** (204 mg, 84%) as an oil. LR-MS: *m/z* = 497 [C_30_H_34_O_5_Na (M + MeOH + Na)^+^].

**Figure 13:**
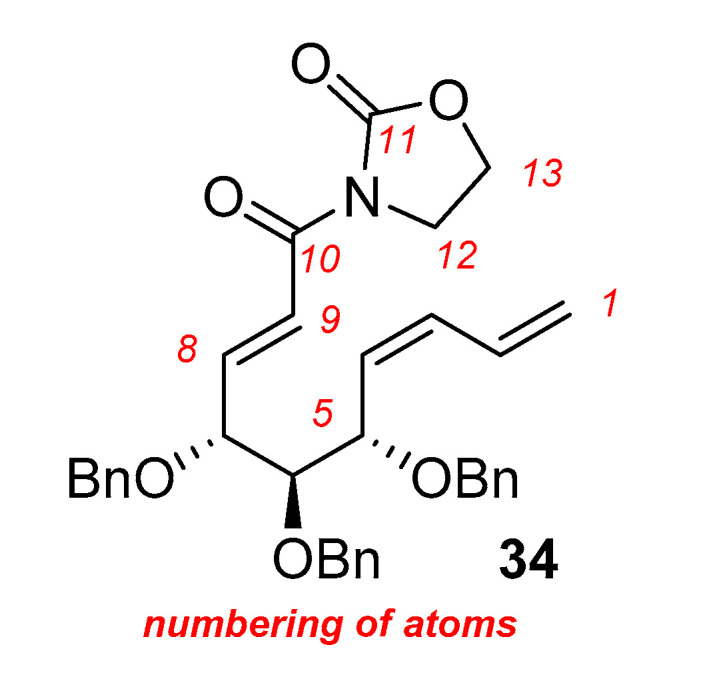
The structure of triene **34** and the numbering of the atoms.

This aldehyde (96 mg, 0.216 mmol) was reacted with phosphonate **23** analogously as for **22**. The crude product was purified column chromatography (hexanes 100%→hexanes-ethyl acetate: 90:10→20:80) to give the title triene **34** as a colorless oil (87.3 mg, 73%) (Figure 13).

^1^H NMR δ: 7.46 (d, *J* = 15.9, 1H, H-9), 7.35–7.16 (m, 16H, arom. and H-8), 6.51 (ddd, *J* = 16.7, 11.3 and 10.1 Hz, 1H, H-2), 6.27 (t, *J* = 11.2 Hz, 1H, H-3), 5.45 (dd, *J* = 11.1 and 9.7 Hz, 1H, H-4), 5.25 (d, *J* = 16.7, 1H, H-1), 5.09 (d, *J* = 10.1 Hz, 1H, H-1′), 4.74 (d, *J* = 11.5 Hz, 1H, OC*H*_2_Ph), 4.67 (d, *J* = 11.3 Hz, 1H, OC*H*_2_Ph), 4.58–4.30 (m, 6H, H-5, H-12, H-12′ and 4×OC*H*_2_Ph), 3.97 (m, 2H, H-13 and H-13′), 3.81−3.74 (m, 2H, H-6 and H-7);

^13^C NMR δ: 164.3 (C-10), 153.1 (C-11), 147.4 (C-8), 138.9, 138.7 and 138.1 (quat. benzyl), 134.0 (C-3), 132.1 (C-2), 128.5−127.2 (arom. and C-4), 122.5 (C-9), 120.1 (C-1), 82.0 and 79.8 (C-6 and C-7), 75.5 (×O*C*H_2_Ph), 75.0 (C-5), 72.4 and 70.4 (2×O*C*H_2_Ph), 62.1 (C-12), 42.7 (C-13).

HR-MS: *m/z* = 553.2456; calcd for C_34_H_35_NO_6_Na [M + Na]^+^: 553.2464.

#### 3.1.13. Bicyclic Compound **35**

**Figure 14:**
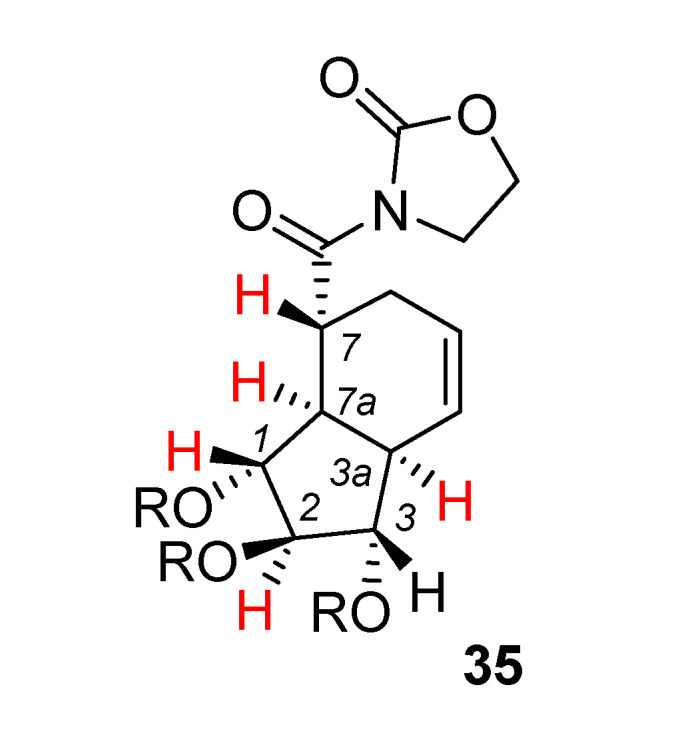
The structure of bicyclic compound **35** and the numbering of the atoms.

To a cooled to −30 °C solution of **34** (39.6 mg, 7.86–10^−2^ mmol) in dry CH_2_Cl_2_ (1.0 mL) a 1.0 M solution of Me_2_AlCl (118 μL, 0.118 mmol, 1.5 eq.) was carefully added and the mixture was stirred for 5 h. Then the mixture was concentrated and the crude product was purified by preparative TLC (*n*-heptane-MTBE: 6:4) to give the cyclic product **35** as colorless oil (26.9 mg, 68%) (Figure 14).

^1^H NMR δ: 7.44–7.16 (m, 15H, arom.), 5.82–5.76 (m, 1H, H-4), 5.68–5.62 (m, 1H, H-5), 4.69–4.58 (m, 4H, 4×OC*H*_2_Ph), 4.59–4.51 (m, 2H, 2×OC*H*_2_Ph), 4.34 (td, *J* = 9.0 and 6.8 Hz, 1H, H-10), 4.28 (td, *J* = 9.1 and 7.0 Hz, 1H, H-10′), 4.03 (t, *J* = 5.0 Hz, 1H, H-2), 3.99–3.85 (m, 3H, H-7, H-11 and H-11′), 3.75 (t, *J* = 5.5 Hz, 1H, H-3), 3.60 (t, *J* = 5.6 Hz, 1H, H-1), 2.94 (brs, 1H, H-3a), 2.64 (q, *J* = 7.2 Hz, 1H, H-7a), 2.26 (dtd, *J* = 17.8, 4.2 and 2.0 Hz, 1H, H-6), 2.08 (dtd, *J* = 17.8, 3.8 and 1.9 Hz, 1H, H-6′);

^13^C NMR δ: 175.4 (C-8), 152.8 (C-9), 138.5, 138.4 and 138.3 (quat. benzyl), 128.4−127.5 (arom. and C-4), 124.3 (C-5), 90.3 (C-2), 88.5 (C-3), 83.4 (C-1), 72.0, 71.9 and 71.5 (3×O*C*H_2_Ph), 61.8 (C-10), 42.7 (C-11), 2 × 40.4 (C-3a and C-7a), 36.9 (C-7), 25.8 (C-6).

HR-MS: *m/z* = 553.2478; calcd for C_34_H_35_NO_6_Na [M + Na]^+^: 553.2464.

Configuration was established on the basis of 1D NOE (see Figure 1 in the text).

## 4. Conclusions

We have proposed a convenient and simple route to optically pure bicyclic carbasugar derivatives from D-xylose derivative. The methodology is based on the introduction of the *Z*-diene system at the anomeric position of a sugar and an olefinic unit at the terminal position. The so obtained triene underwent smooth and highly stereoselective cyclization providing the bicyclic derivative a good yield. The analogous triene prepared from the D-glucose derivative did not, however, undergo any cyclization even under high pressure. We do not know yet the reason for such peculiar behavior.

It is very likely that our methodology leading to bicyclic sugar mimetics can be applied to other simple sugars containing 5-carbon atoms in the chain. Very problematic, however, is the application of our methodology to hexoses.

## Supplementary Materials

The supporting information with the scans of the NMR data of all compounds are available online.
