# Supplementary material for: Synthesis of Highly Oxygenated Bicyclic Carbasugars. Remarkable Difference in the Reactivity of the d-gluco and d-xylo- Derived Trienes
Source: Molecules. 2020 Jul 24;25(15):3357. doi: 10.3390/molecules25153357 (PMC7436230; doi:10.3390/molecules25153357)
Supplement: Supplementary file 1 [file molecules-25-03357-s001.pdf]

Supporting information

Synthesis of highly oxygenated bicyclic carbasugars. Remarkable difference in the reactivity of the D-*gluco* and D-*xylo*- derived triens.

Grzegorz Witkowski, Mykhaylo A. Potopnyk, Karolina Tiara, Anna Osuch-Kwiatkowska, and Sławomir Jarosz

Institute of Organic Chemistry, Polish Academy of Sciences, Kasprzaka 44/52, 01-224 Warsaw, Poland

The NMR data (^{1}H, COSY, ^{13}C, HSQC) of key-compounds

**Dienoalcohol 21**	**pages 2-5**
**Triene 24**	**pages 6-9**
**Triene 25**	**pages 10-13**
**Triene 26**	**pages 14-17**
**Dienoalcohol 32**	**pages 18-21**
**Dienoalcohol 32**	**pages 18-21**
**Bicyclic compound 35**	**pages 22-25**

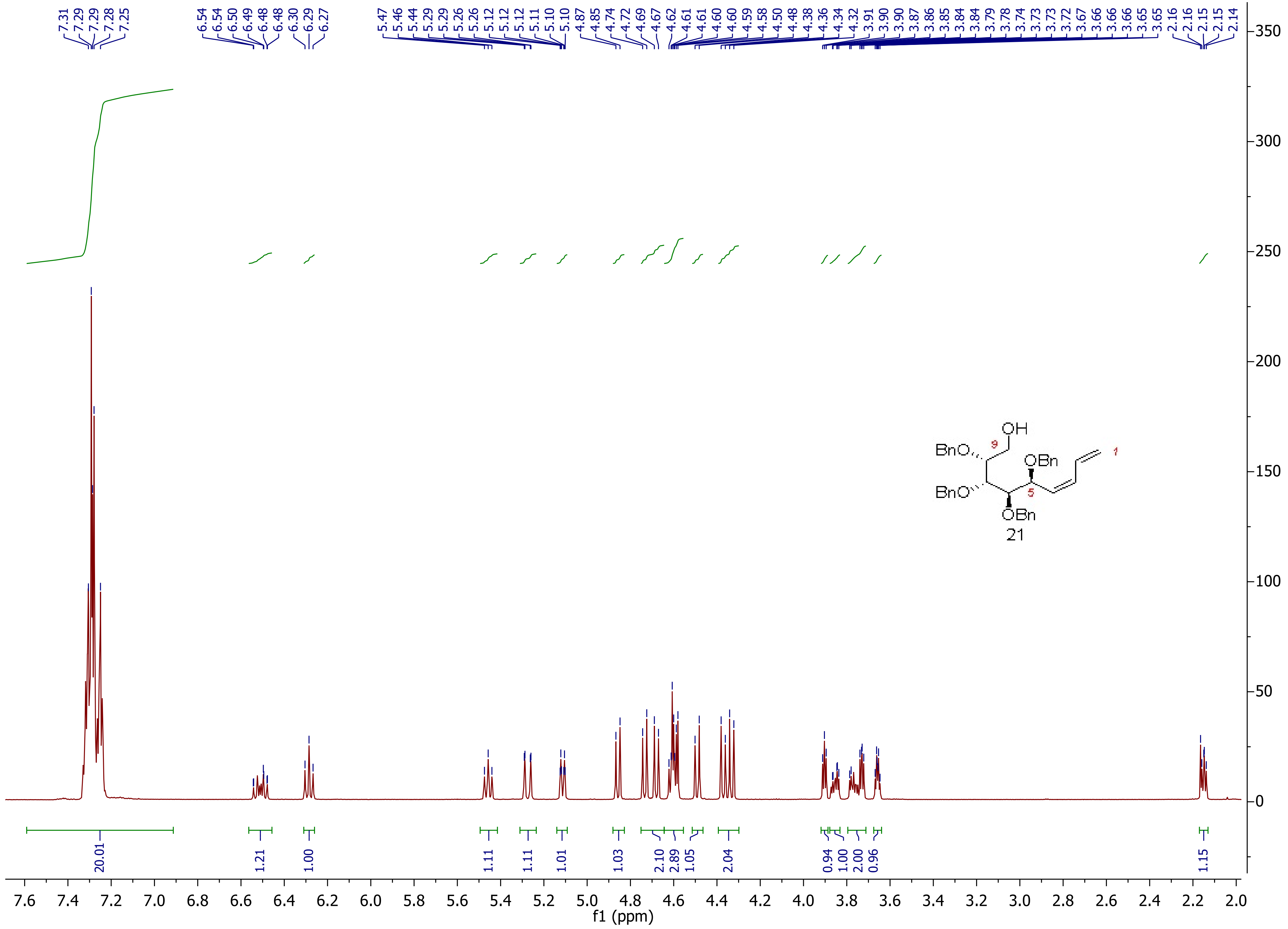

7.31 7.29 7.29 7.28 7.25
6.54 6.54 6.50 6.49 6.48 6.48 6.30 6.29 6.27
5.47 5.46 5.44 5.29 5.29 5.26 5.26 5.12 5.12 5.12 5.11 5.10 5.10 4.87 4.85 4.74 4.72 4.69 4.67 4.62 4.61 4.61 4.60 4.60 4.59 4.58 4.50 4.48 4.38 4.36 4.34 4.32 3.91 3.90 3.90 3.87 3.86 3.85 3.84 3.84 3.79 3.78 3.74 3.73 3.73 3.72 3.67 3.66 3.66 3.66 3.65 3.65
2.16 2.16 2.15 2.15 2.14
20.01 1.21 1.00 1.11 1.11 1.01 1.03 2.10 2.89 1.05 2.04 0.94 1.00 2.00 0.96 1.15
OH
BnO
OBn
BnO
OBn
9
5
1
21
f1 (ppm)

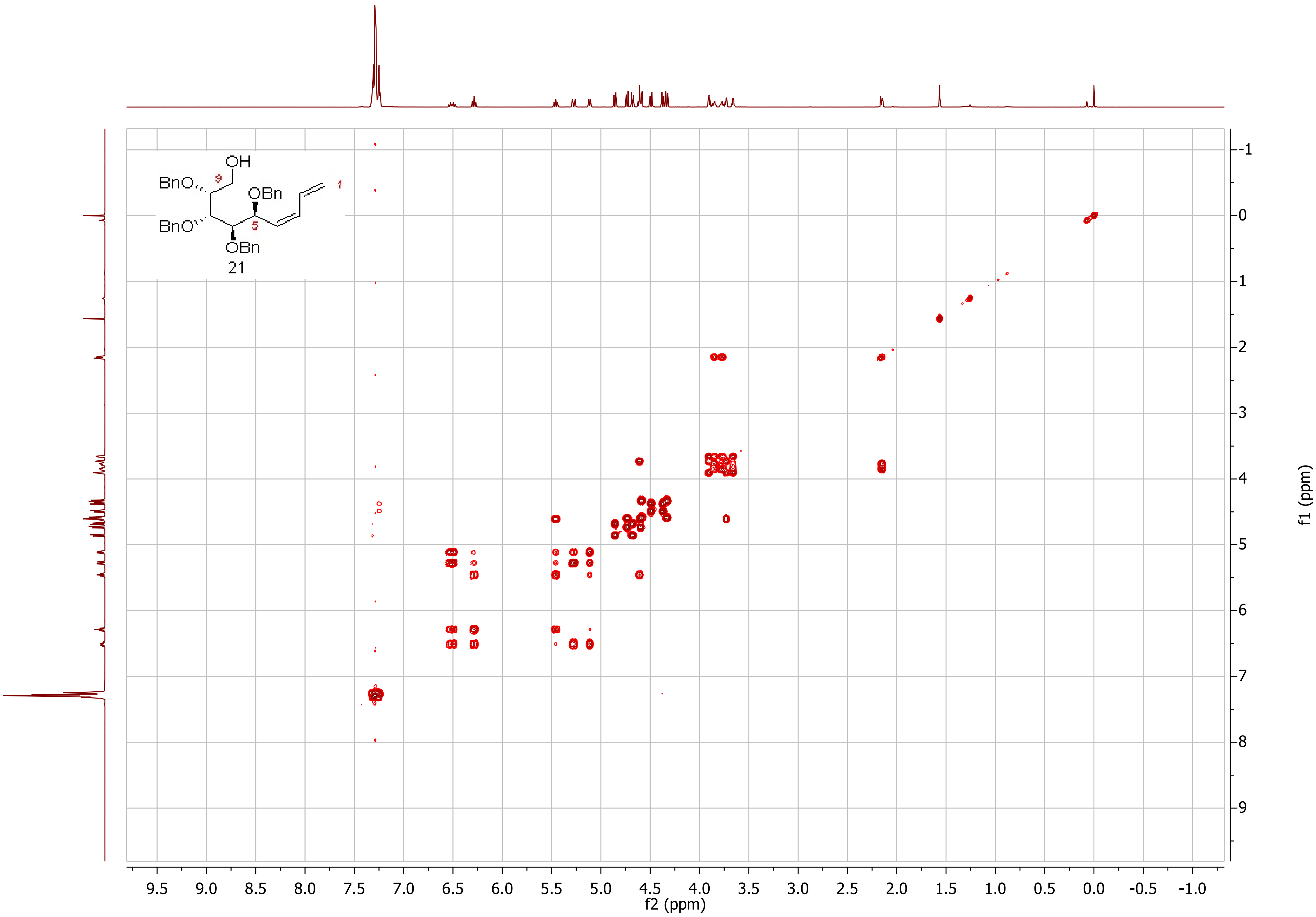

OH
BnO
9
OBn
1
BnO
5
OBn
21
f2 (ppm)
f1 (ppm)

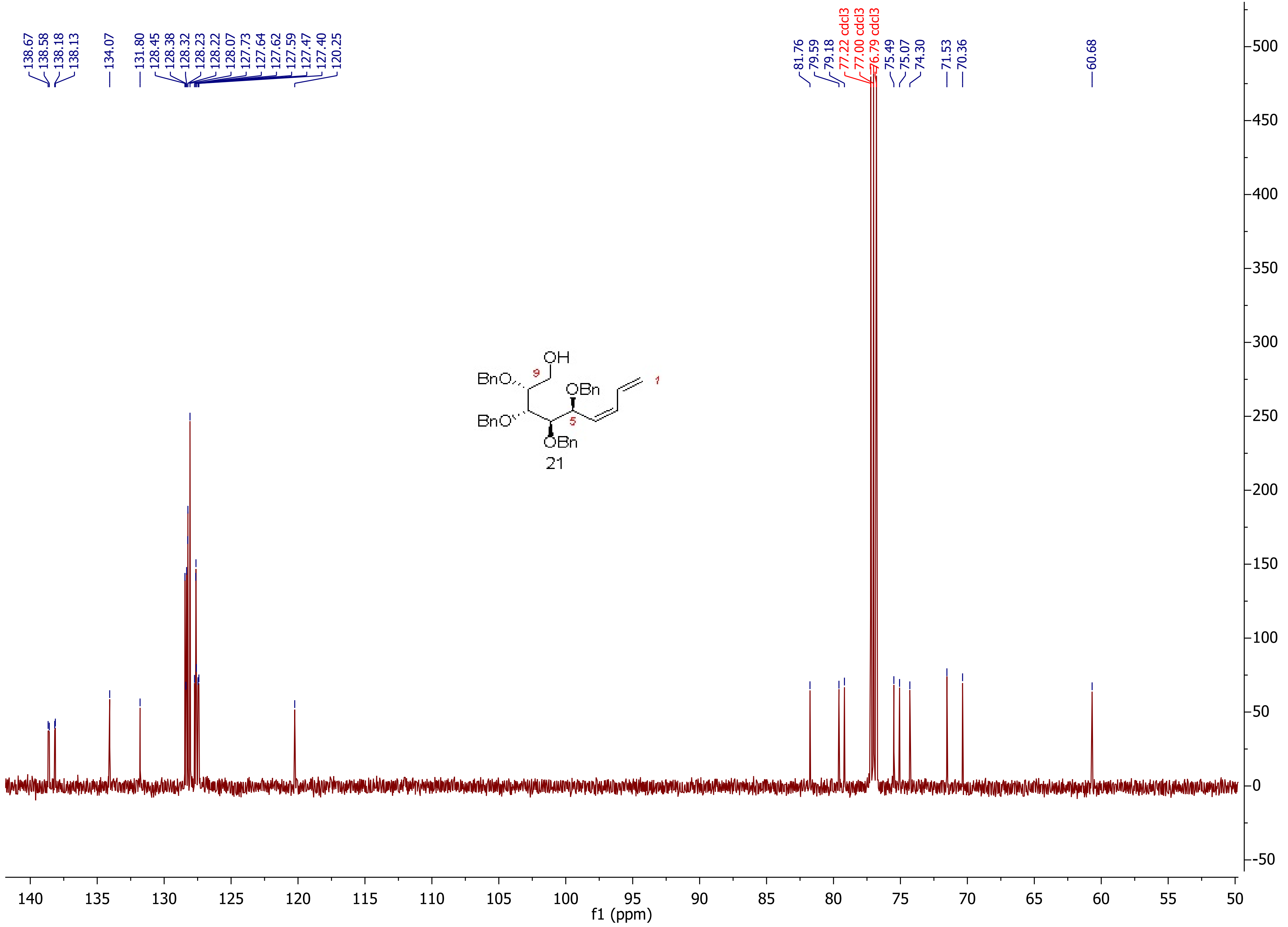

138.67
138.58
138.18
138.13
134.07
131.80
128.45
128.38
128.32
128.23
128.22
128.07
127.73
127.64
127.62
127.59
127.47
127.40
120.25
81.76
79.59
79.18
77.22 cdcl3
77.00 cdcl3
76.79 cdcl3
75.49
75.07
74.30
71.53
70.36
60.68
OH
BnO
OBn
BnO
OBn
9
5
1
21
f1 (ppm)

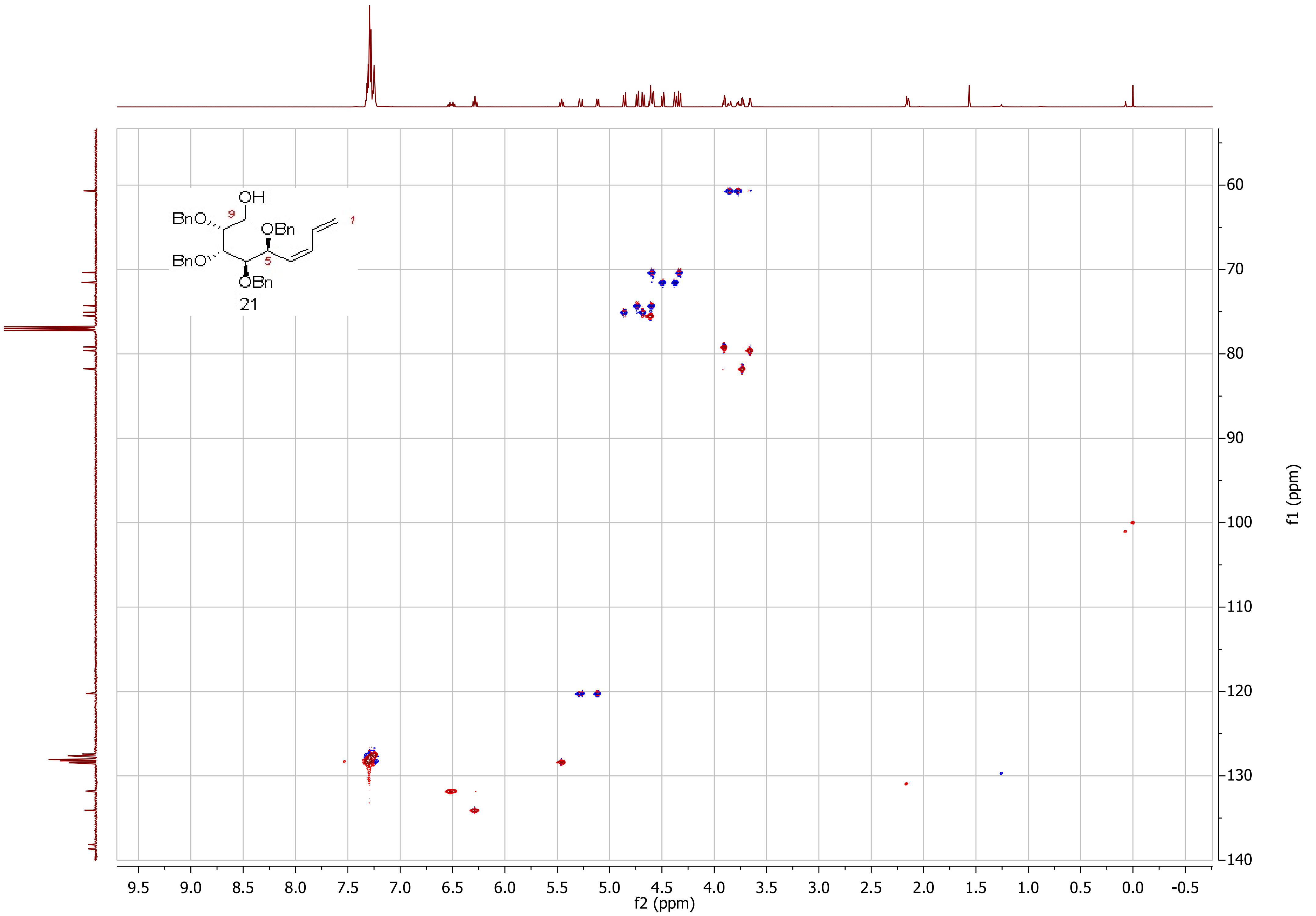

OH
BnO
9
OBn
1
BnO
5
OBn
21
f2 (ppm)
f1 (ppm)

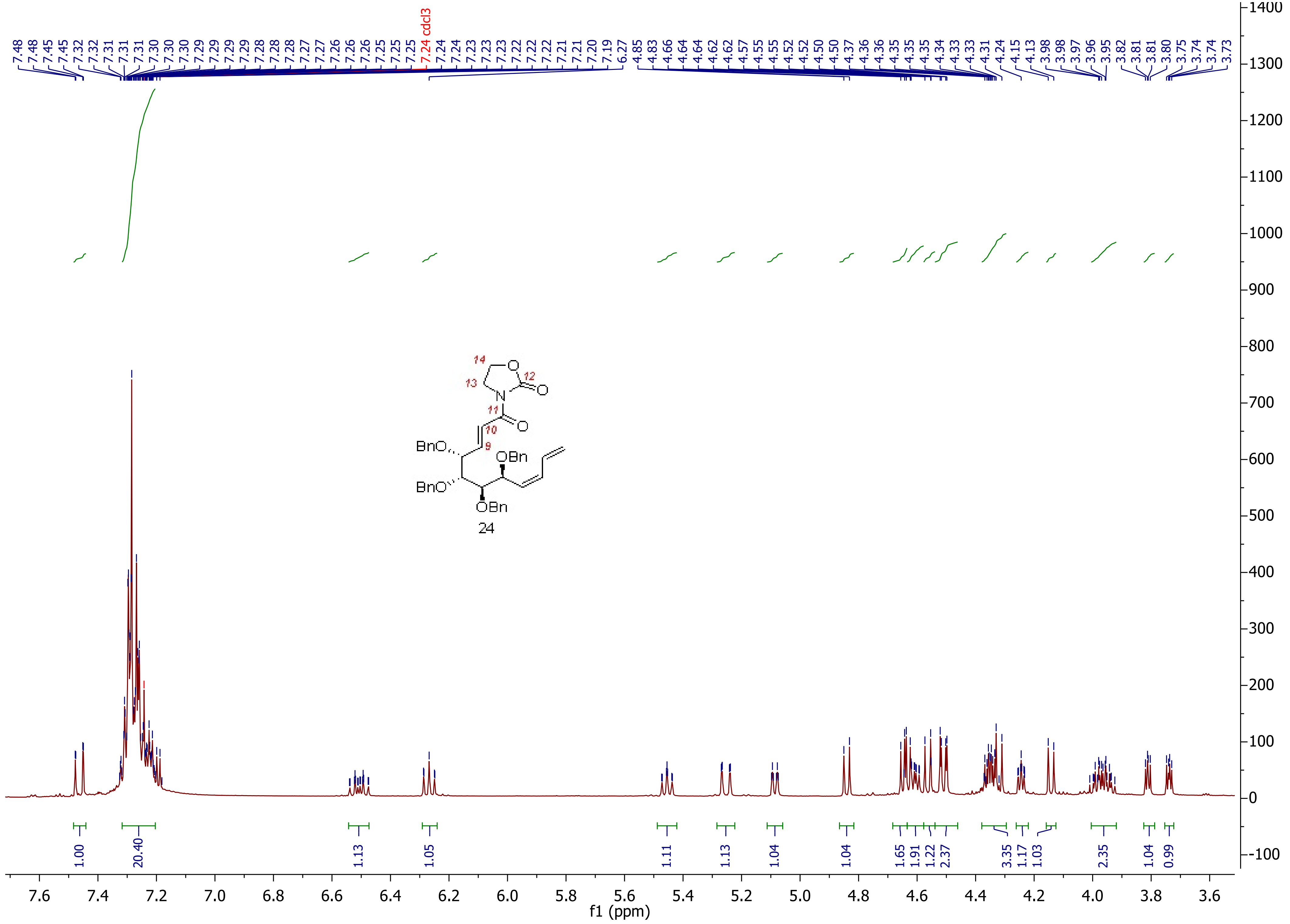

7.48 7.48 7.45 7.45 7.32 7.32 7.31 7.31 7.31 7.30 7.30 7.29 7.29 7.29 7.29 7.28 7.28 7.28 7.27 7.27 7.26 7.26 7.25 7.25 7.25 7.24 cdcl3 7.24 7.24 7.23 7.23 7.23 7.22 7.22 7.22 7.21 7.21 7.20 7.19 6.27 4.85 4.83 4.66 4.64 4.62 4.62 4.57 4.55 4.55 4.52 4.52 4.50 4.50 4.37 4.36 4.36 4.35 4.35 4.35 4.34 4.33 4.33 4.31 4.24 4.15 4.13 3.98 3.98 3.97 3.96 3.95 3.82 3.81 3.81 3.80 3.75 3.74 3.74 3.73
14
13
12
11
10
9
O
N
BnO
BnO
OBn
OBn
24
1.00 20.40 1.13 1.05 1.11 1.13 1.04 1.04 1.65 1.91 1.22 2.37 3.35 1.17 1.03 2.35 1.04 0.99
f1 (ppm)

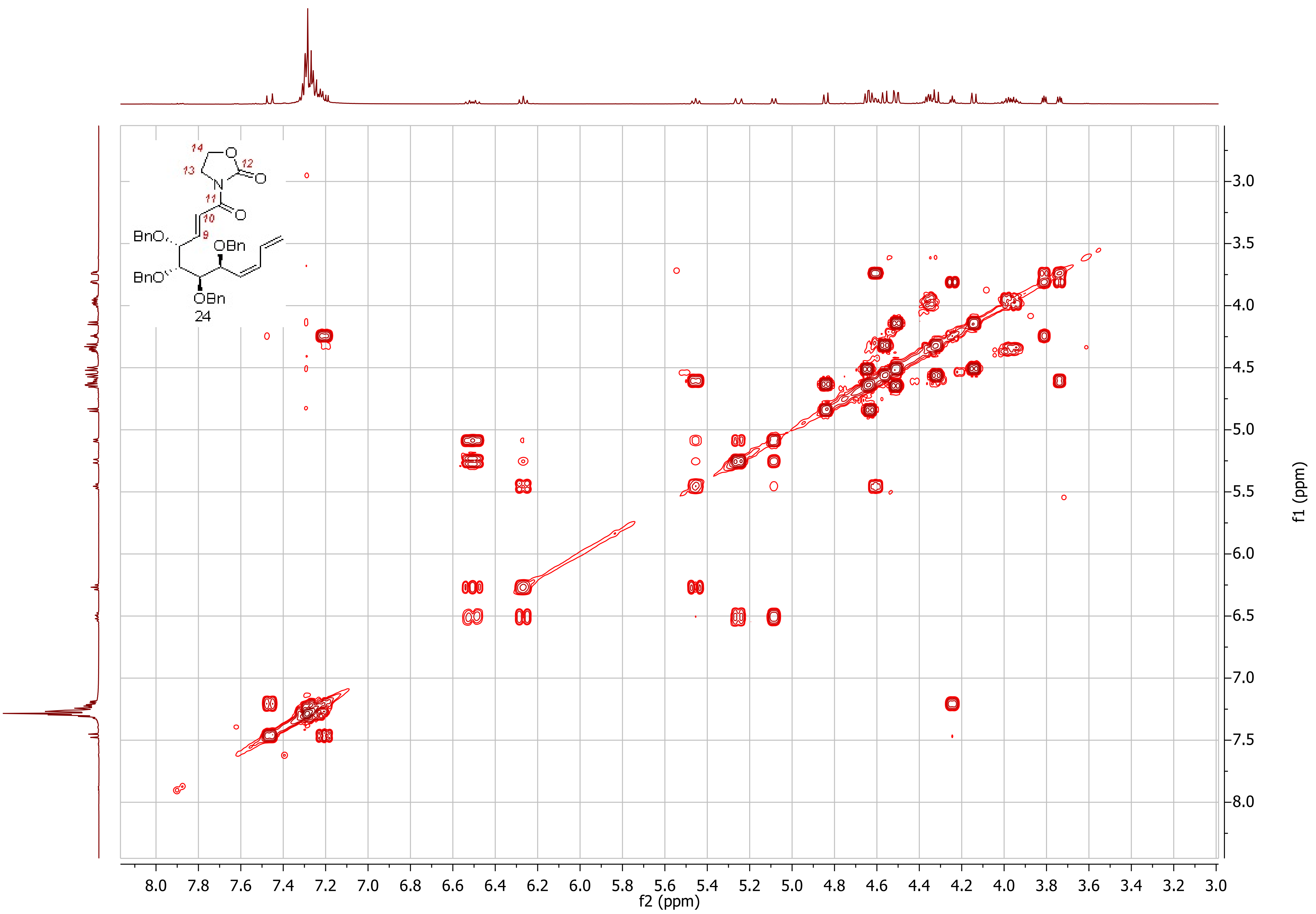

14
13
12
11
10
9
N
O
BnO
OBn
BnO
OBn
24
f2 (ppm)
f1 (ppm)

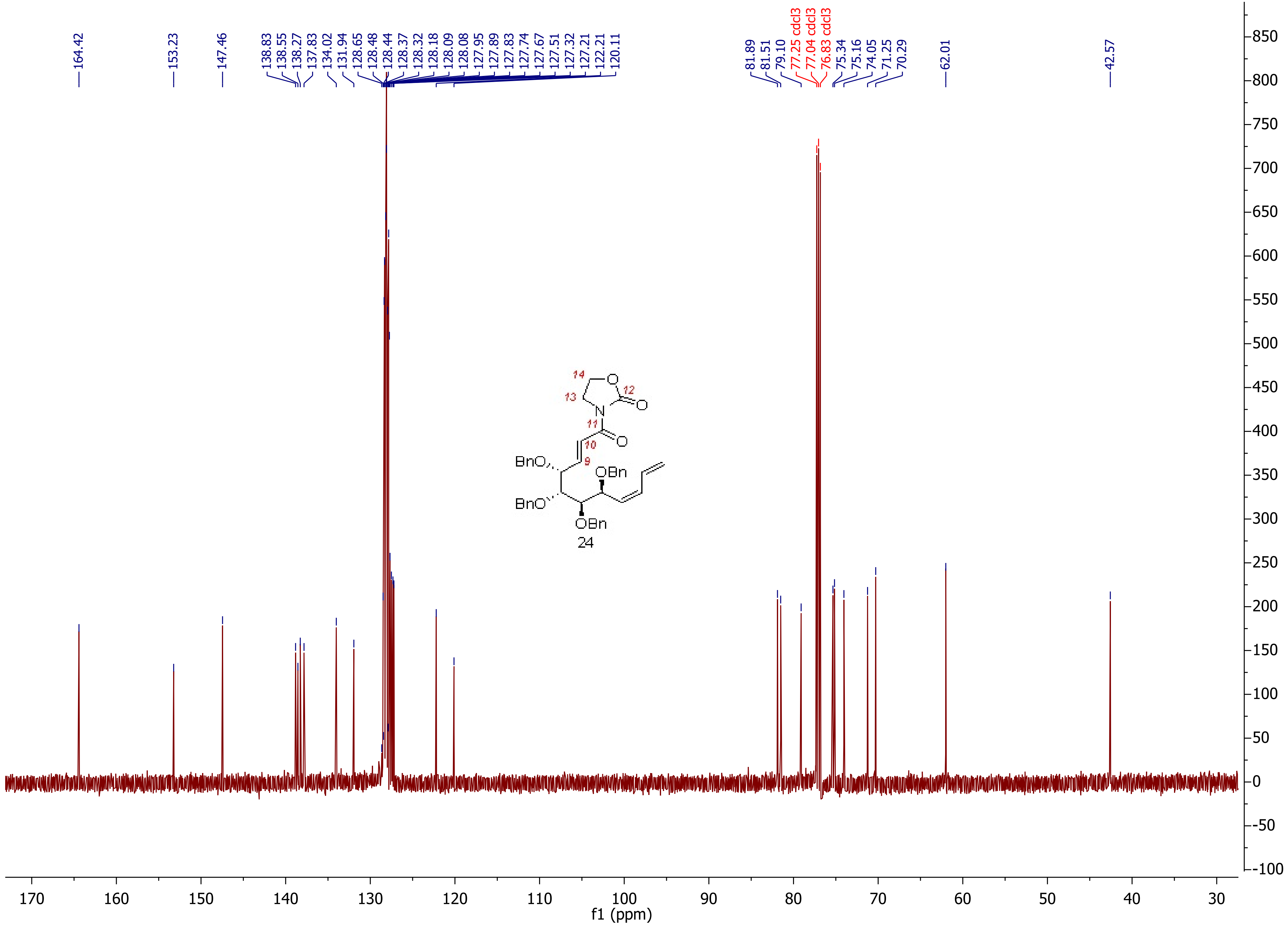

164.42
153.23
147.46
138.83
138.55
138.27
137.83
134.02
131.94
128.65
128.48
128.44
128.37
128.32
128.18
128.09
128.08
127.95
127.89
127.83
127.74
127.67
127.51
127.32
127.21
122.21
120.11
81.89
81.51
79.10
77.25 cdcl3
77.04 cdcl3
76.83 cdcl3
75.34
75.16
74.05
71.25
70.29
62.01
42.57
14
13
12
11
10
9
O
N
BnO
OBn
24
f1 (ppm)

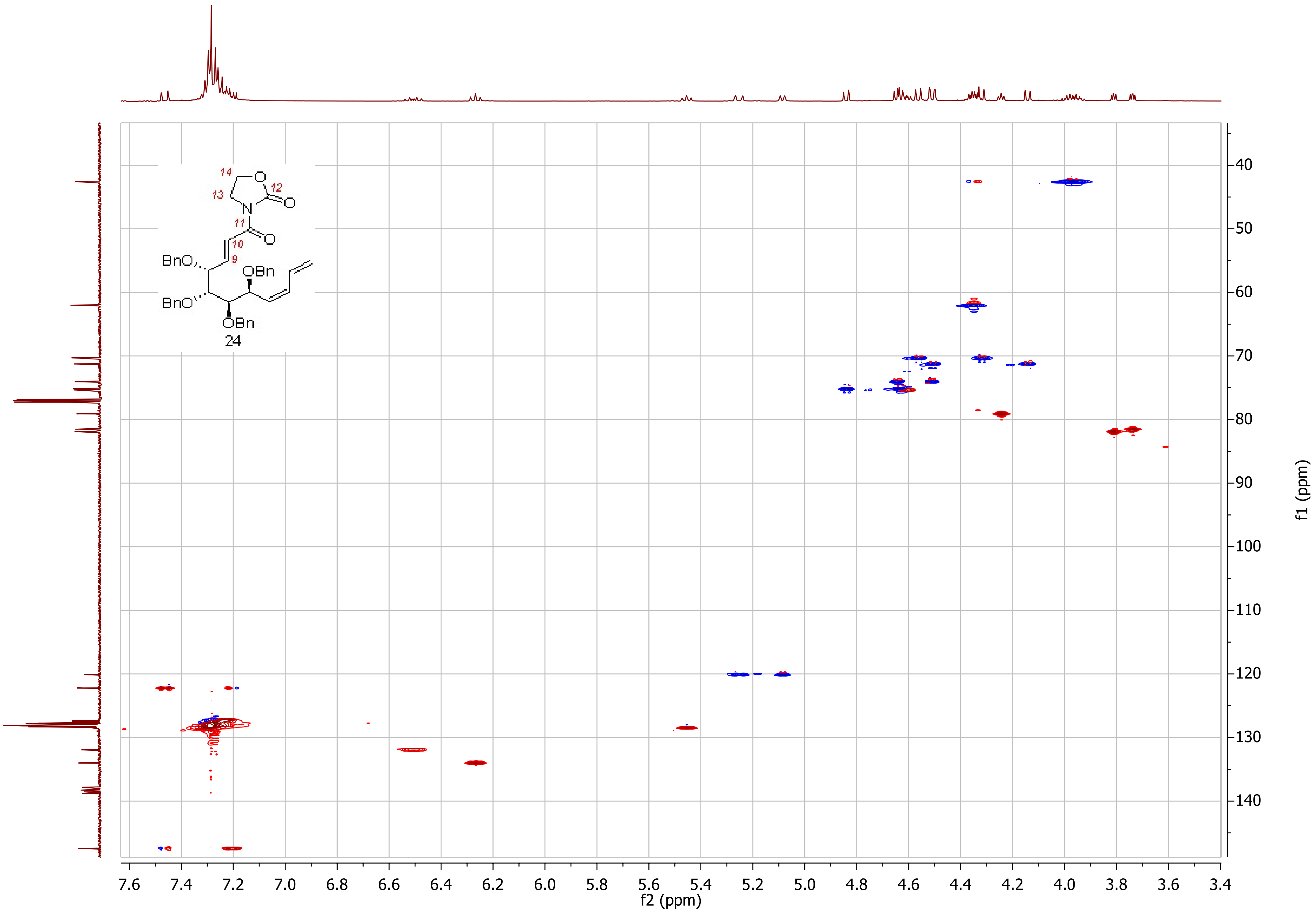

BnO
OBn
BnO
OBn
24
7.6
7.4
7.2
7.0
6.8
6.6
6.4
6.2
6.0
5.8
5.6
5.4
5.2
5.0
4.8
4.6
4.4
4.2
4.0
3.8
3.6
3.4
f2 (ppm)
40
50
60
70
80
90
100
110
120
130
140
f1 (ppm)

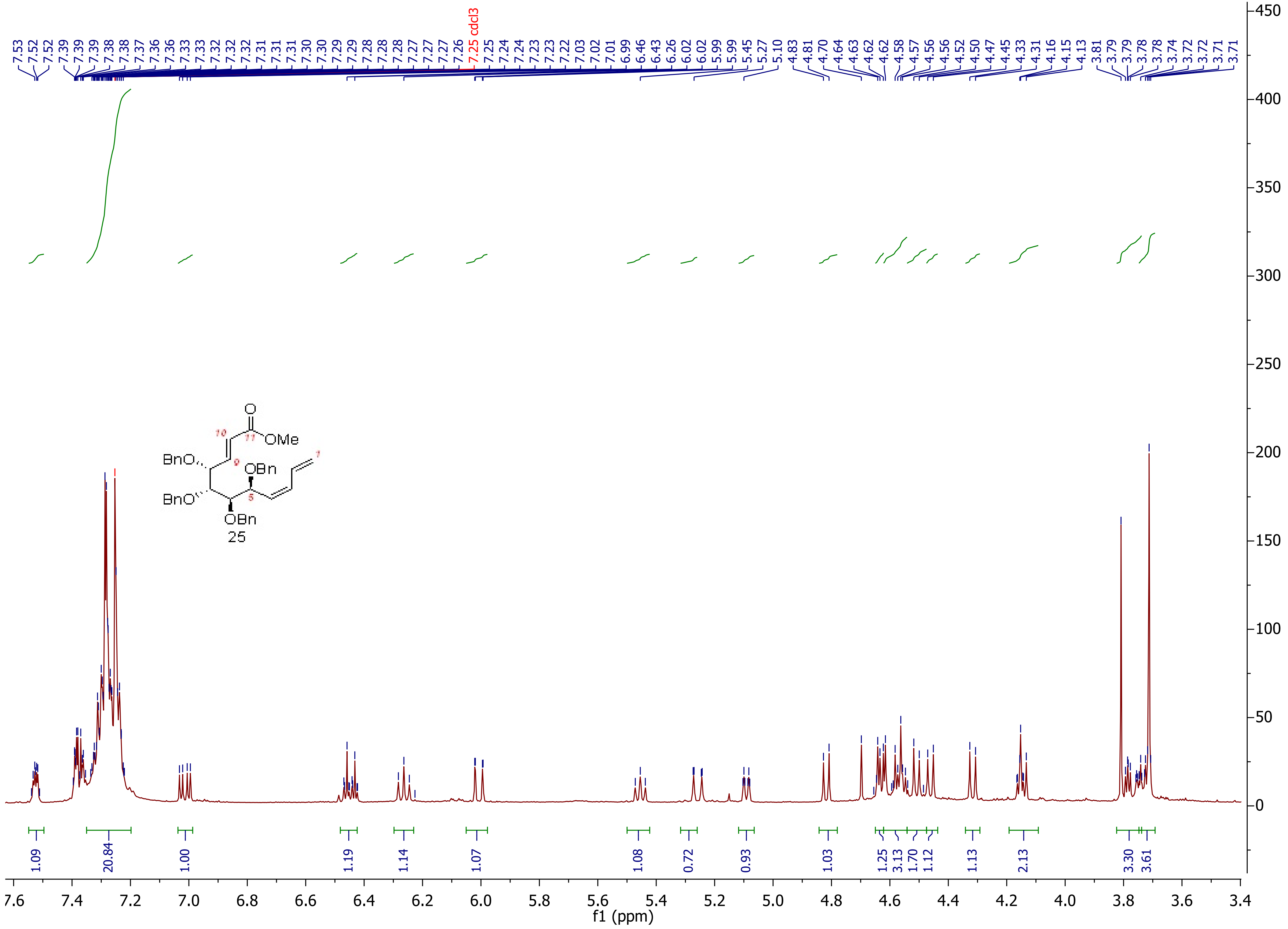
7.53 7.52 7.52 7.39 7.39 7.39 7.38 7.38 7.37 7.36 7.36 7.33 7.33 7.32 7.32 7.32 7.31 7.31 7.31 7.30 7.30 7.29 7.29 7.28 7.28 7.28 7.27 7.27 7.27 7.26 7.25 cdcl3 7.25 7.24 7.24 7.23 7.23 7.22 7.03 7.02 7.01 6.99 6.46 6.43 6.26 6.02 6.02 5.99 5.99 5.45 5.27 5.10 4.83 4.81 4.70 4.64 4.63 4.62 4.62 4.58 4.57 4.56 4.56 4.52 4.50 4.47 4.45 4.33 4.31 4.16 4.15 4.13 3.81 3.79 3.79 3.78 3.78 3.74 3.72 3.72 3.71 3.71
O
OMe
BnO
OBn
BnO
OBn
25
1.09 20.84 1.00 1.19 1.14 1.07 1.08 0.72 0.93 1.03 1.25 3.13 1.70 1.12 1.13 2.13 3.30 3.61
7.6 7.4 7.2 7.0 6.8 6.6 6.4 6.2 6.0 5.8 5.6 5.4 5.2 5.0 4.8 4.6 4.4 4.2 4.0 3.8 3.6 3.4
f1 (ppm)
450 400 350 300 250 200 150 100 50 0

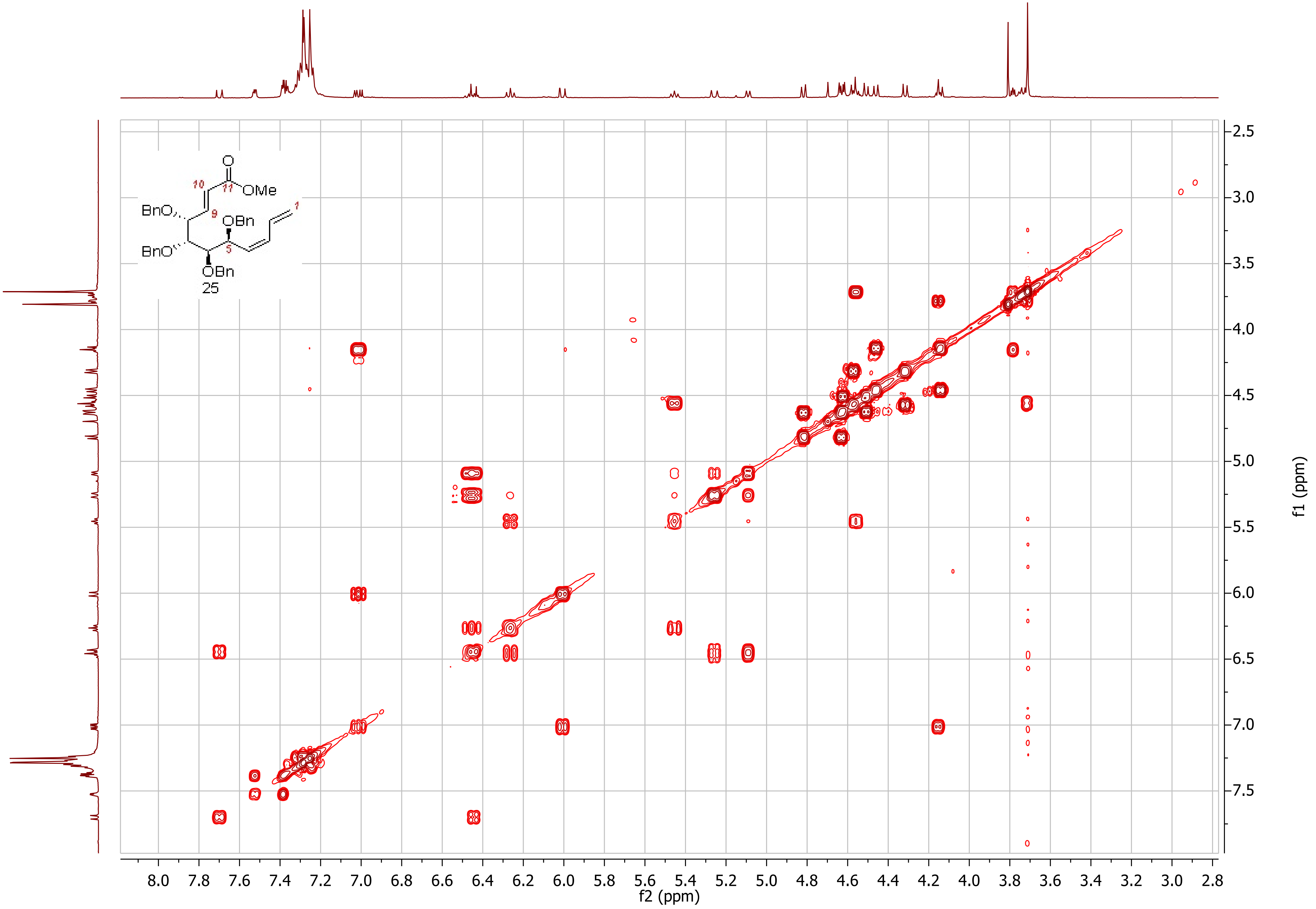

O
OMe
BnO
BnO
OBn
OBn
25
f2 (ppm)
f1 (ppm)
8.0
7.8
7.6
7.4
7.2
7.0
6.8
6.6
6.4
6.2
6.0
5.8
5.6
5.4
5.2
5.0
4.8
4.6
4.4
4.2
4.0
3.8
3.6
3.4
3.2
3.0
2.8
2.5
3.0
3.5
4.0
4.5
5.0
5.5
6.0
6.5
7.0
7.5

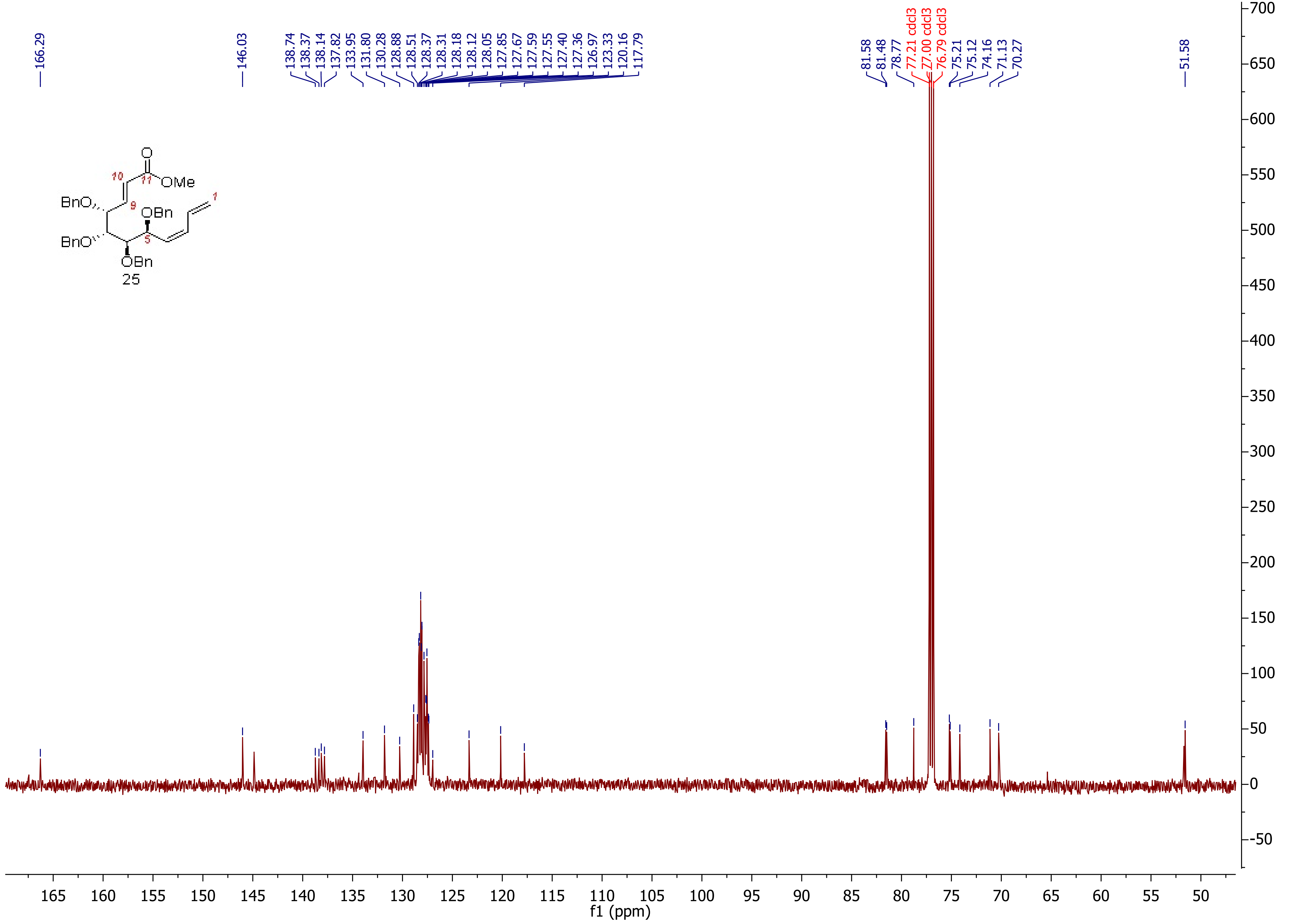
166.29
146.03
138.74
138.37
138.14
137.82
133.95
131.80
130.28
128.88
128.51
128.37
128.31
128.18
128.12
128.05
127.85
127.67
127.59
127.55
127.40
127.36
126.97
123.33
120.16
117.79
81.58
81.48
78.77
77.21 cdcl3
77.00 cdcl3
76.79 cdcl3
75.21
75.12
74.16
71.13
70.27
51.58
OMe
BnO
OBn
25
f1 (ppm)

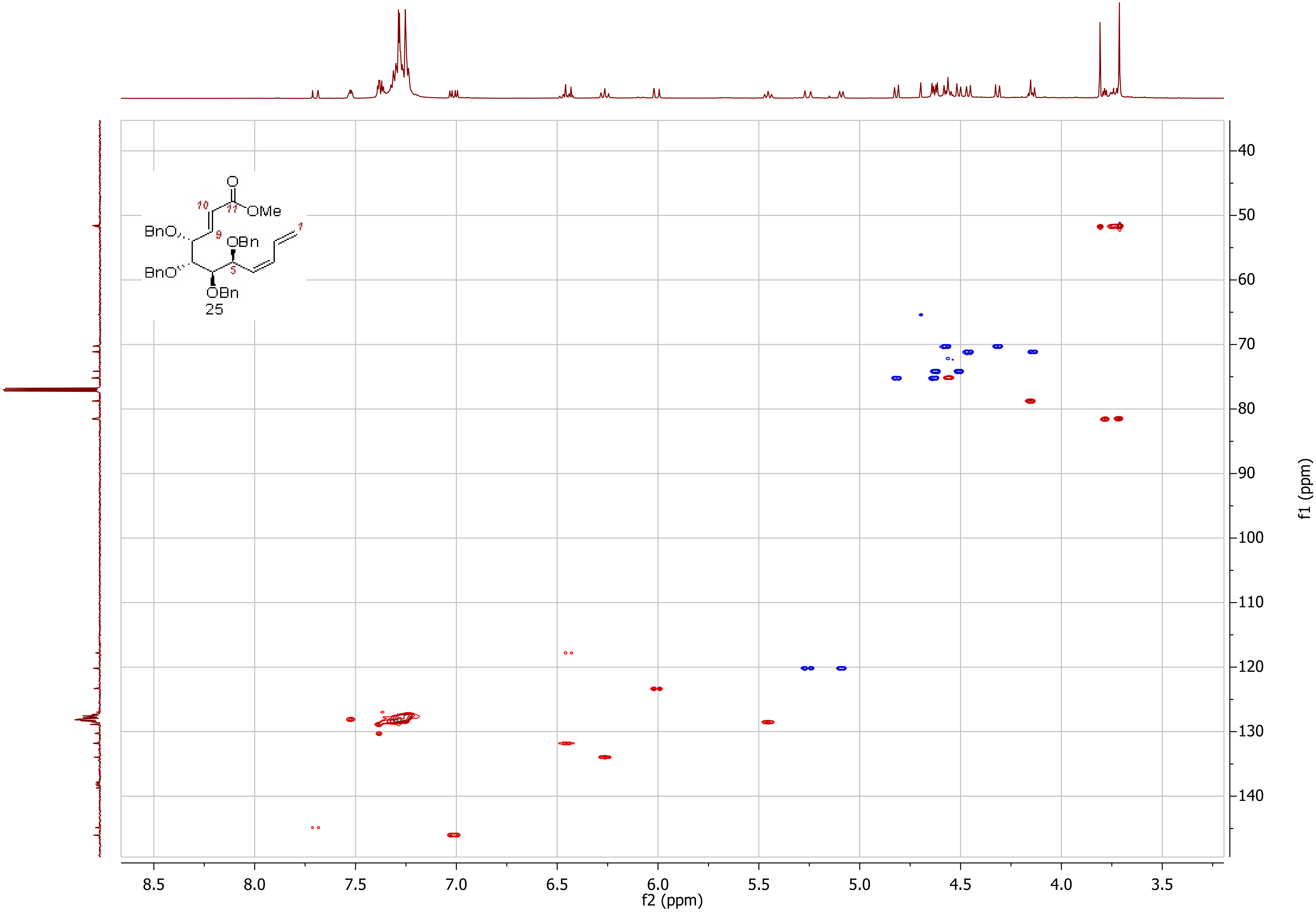

BnO
BnO
OBn
OBn
OMe
O
25
f2 (ppm)
f1 (ppm)

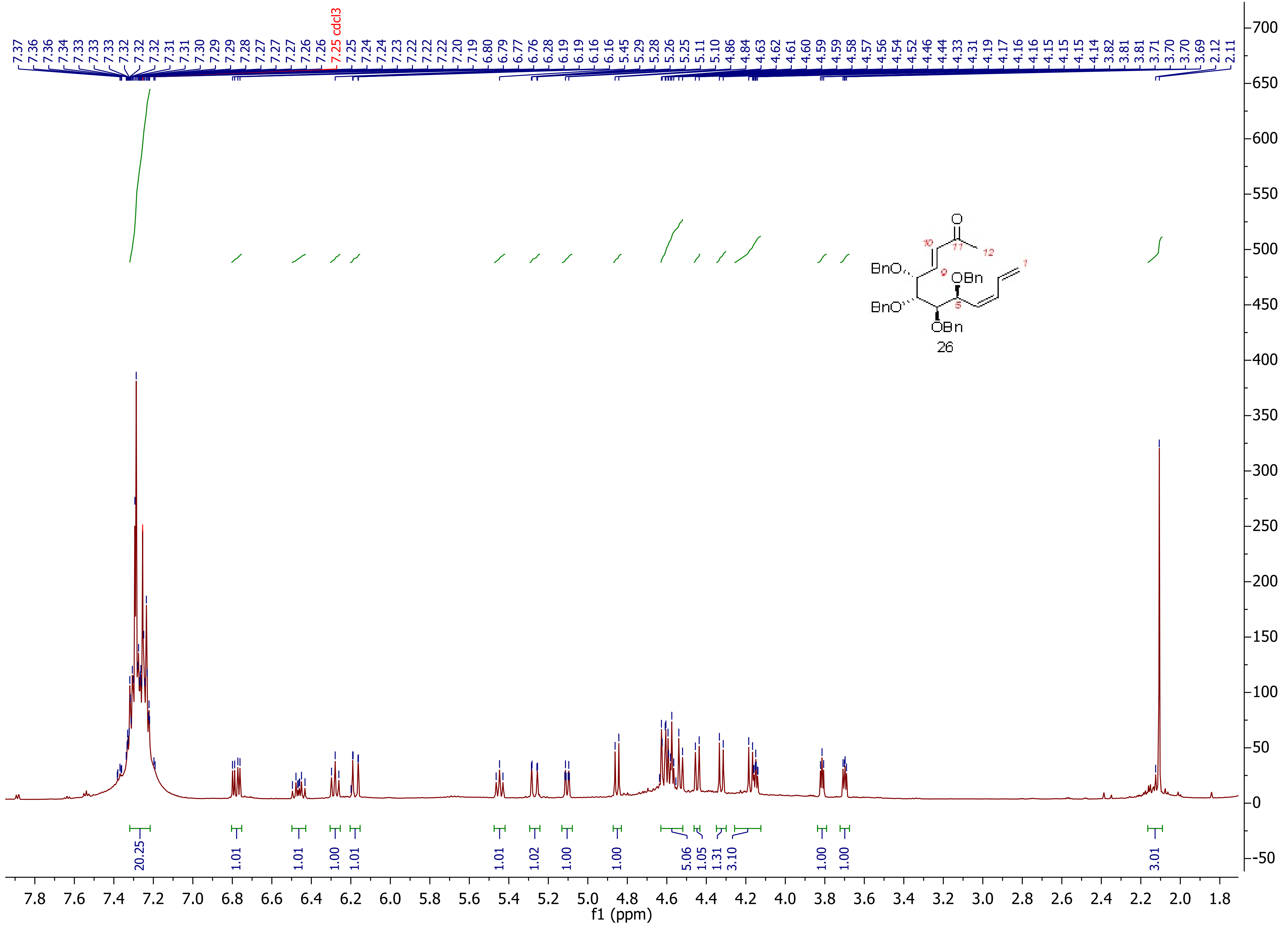
f1 (ppm)
26
BnO
OBn
cdcl3

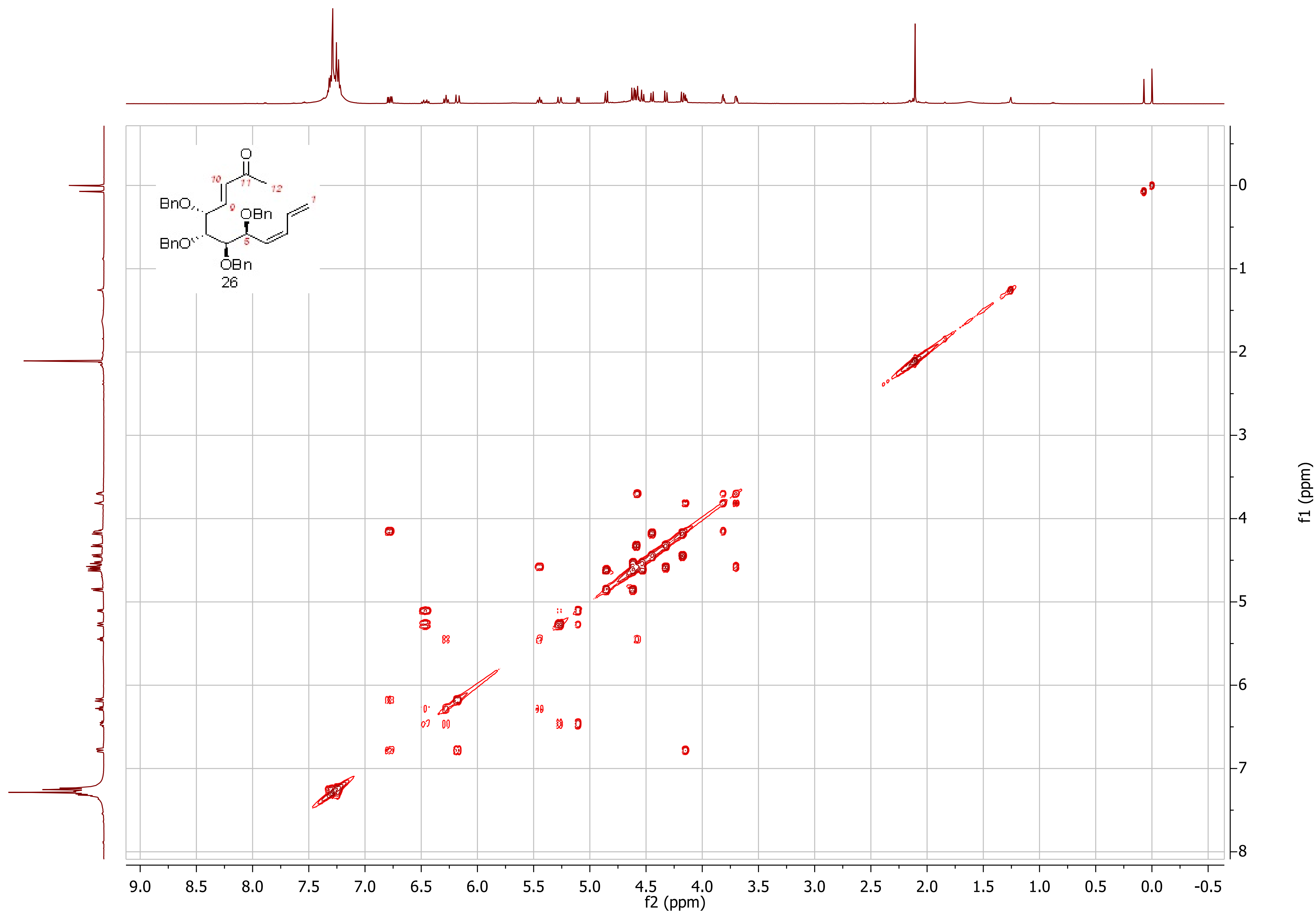

BnO
BnO
OBn
OBn
O
26
f2 (ppm)
f1 (ppm)

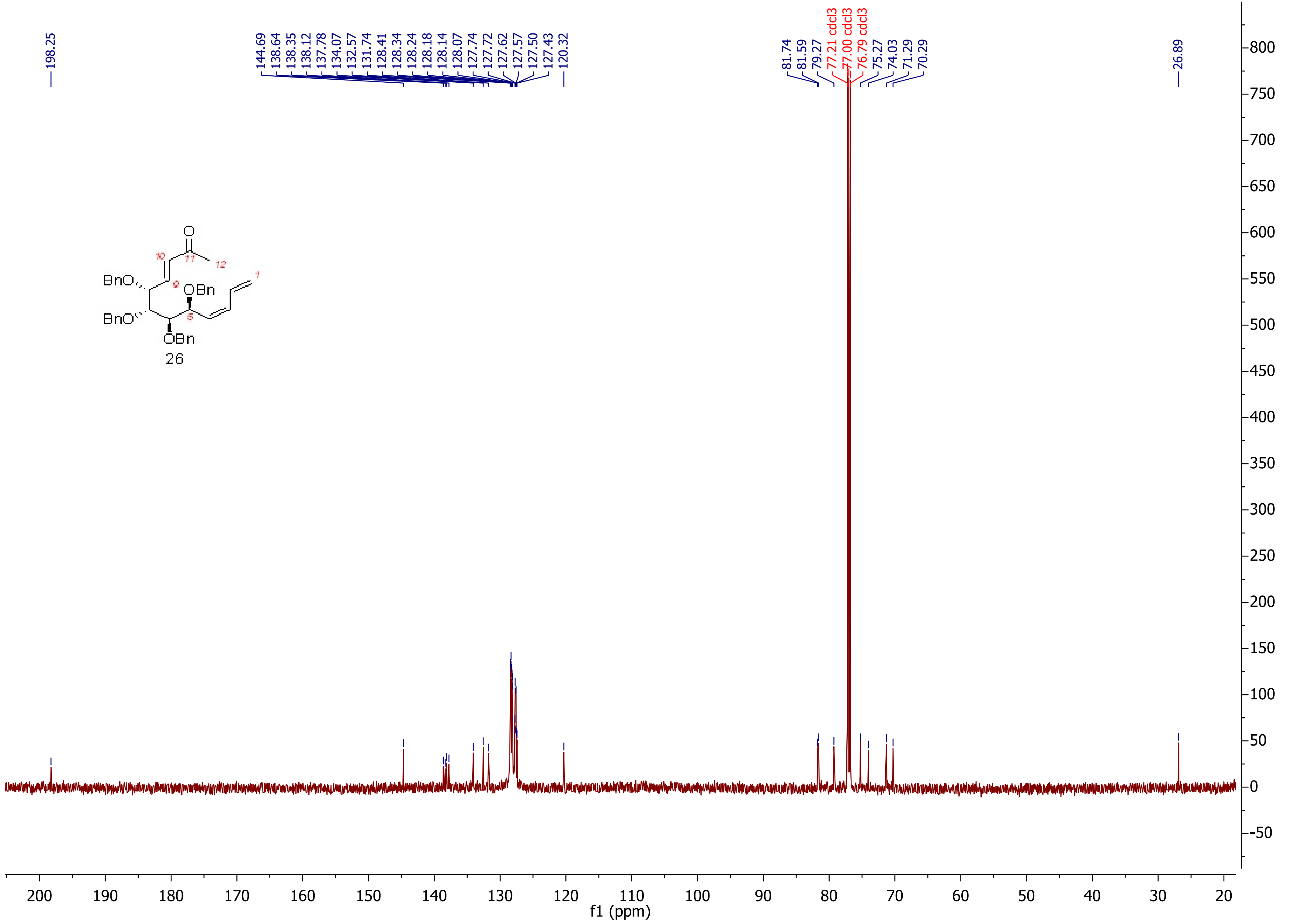
198.25
144.69
138.64
138.35
138.12
137.78
134.07
132.57
131.74
128.41
128.34
128.24
128.18
128.14
128.07
127.74
127.72
127.62
127.57
127.50
127.43
120.32
81.74
81.59
79.27
77.21 cdcl3
77.00 cdcl3
76.79 cdcl3
75.27
74.03
71.29
70.29
26.89
BnO
OBn
26
f1 (ppm)

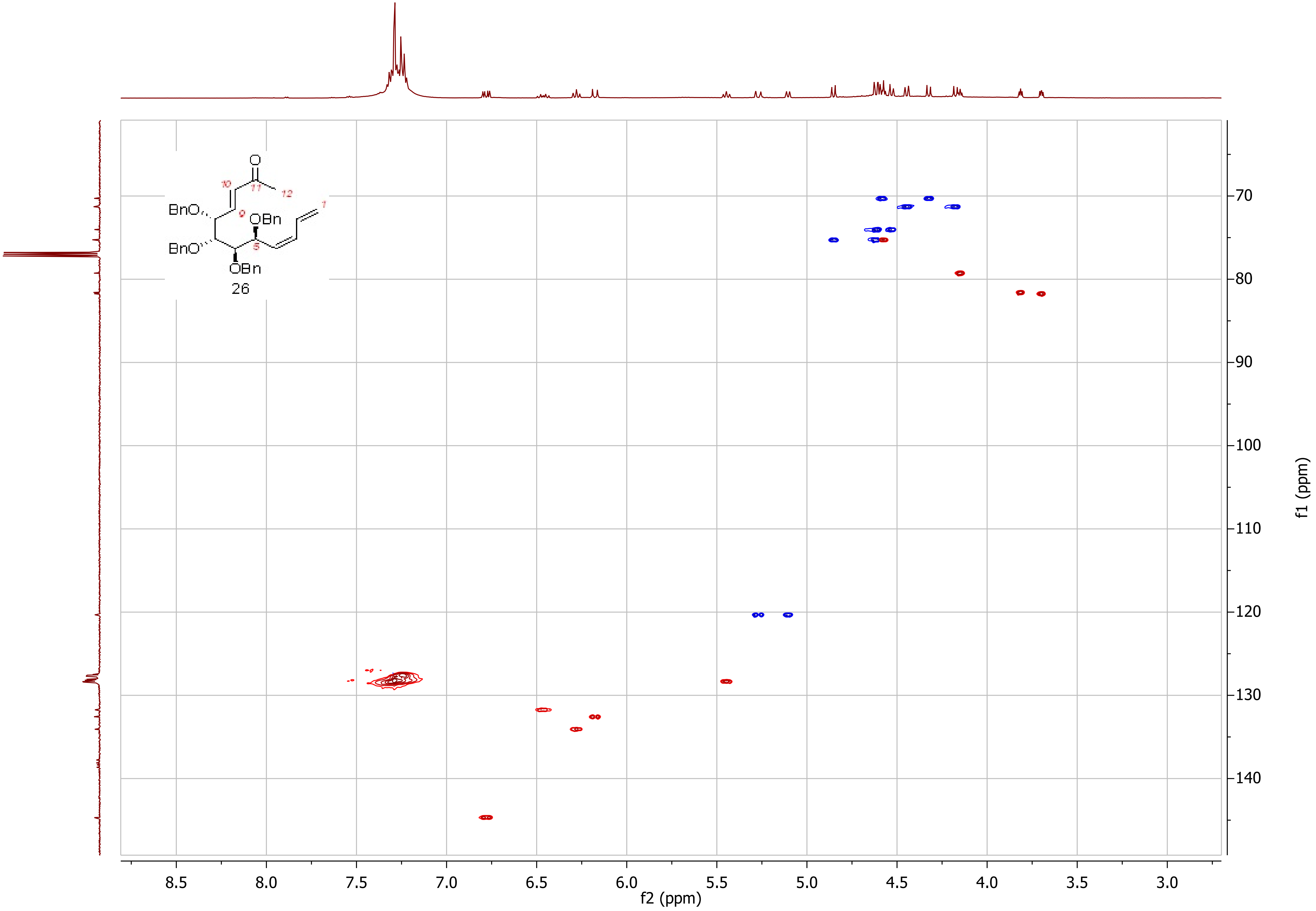

O
BnO
OBn
BnO
OBn
26
10
11
12
9
5
1
70
80
90
100
110
120
130
140
f1 (ppm)
8.5
8.0
7.5
7.0
6.5
6.0
5.5
5.0
4.5
4.0
3.5
3.0
f2 (ppm)

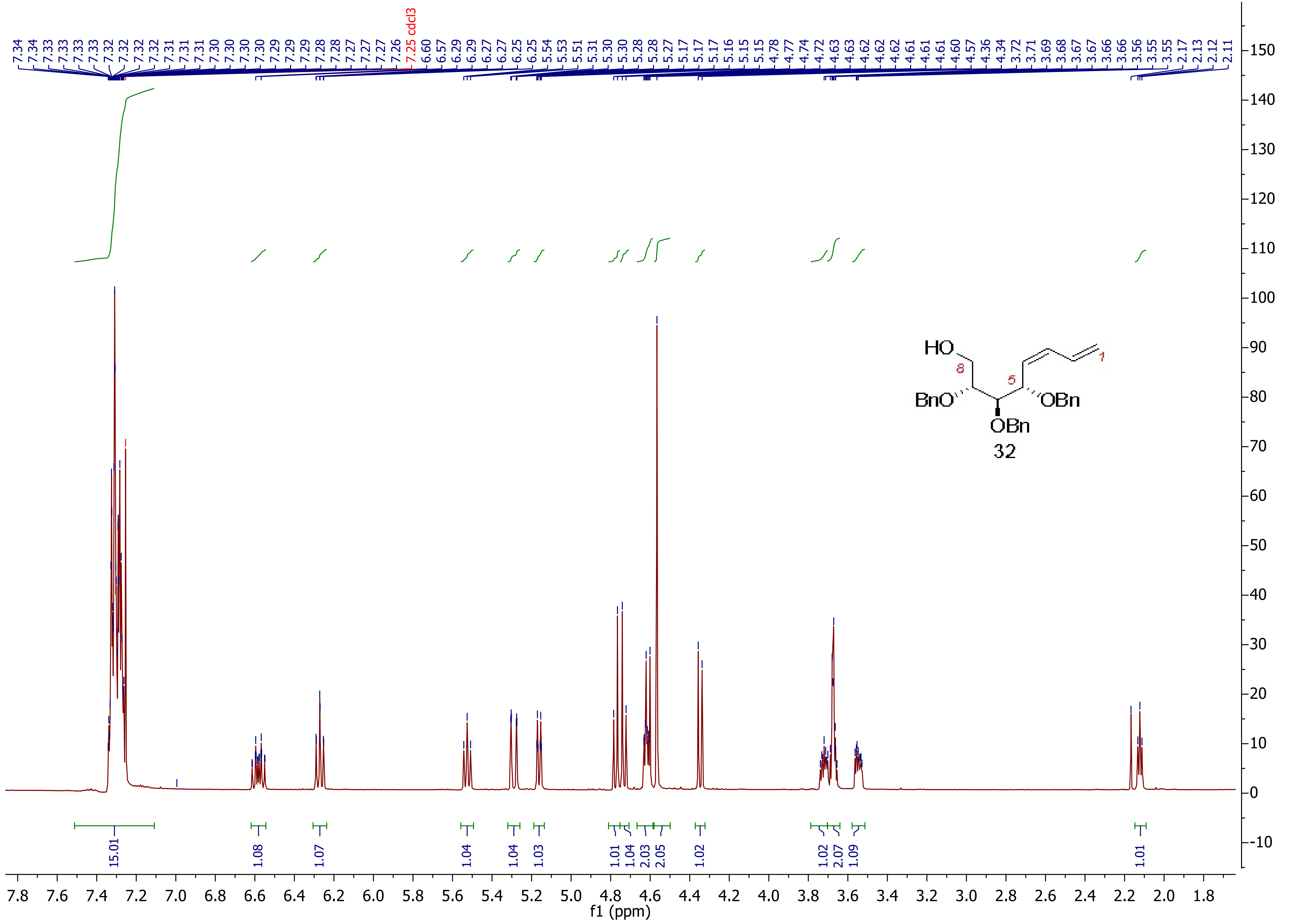
7.34 7.34 7.33 7.33 7.33 7.33 7.32 7.32 7.32 7.32 7.31 7.31 7.31 7.30 7.30 7.30 7.30 7.29 7.29 7.29 7.28 7.28 7.27 7.27 7.27 7.26
7.25 cdcl3
6.60 6.57 6.29 6.29 6.27 6.27 6.25 6.25 5.54 5.53 5.51 5.31 5.30 5.30 5.28 5.28 5.27 5.17 5.17 5.17 5.16 5.15 5.15 4.78 4.77 4.74 4.72 4.63 4.63 4.62 4.62 4.62 4.61 4.61 4.61 4.60 4.57 4.36 4.34 3.72 3.71 3.69 3.68 3.67 3.67 3.66 3.66 3.56 3.55 3.55 2.17 2.13 2.12 2.11
HO
8
5
1
BnO
OBn
OBn
32
15.01
1.08
1.07
1.04
1.04
1.03
1.01
1.04
2.03
2.05
1.02
1.02
2.07
1.09
1.01
f1 (ppm)

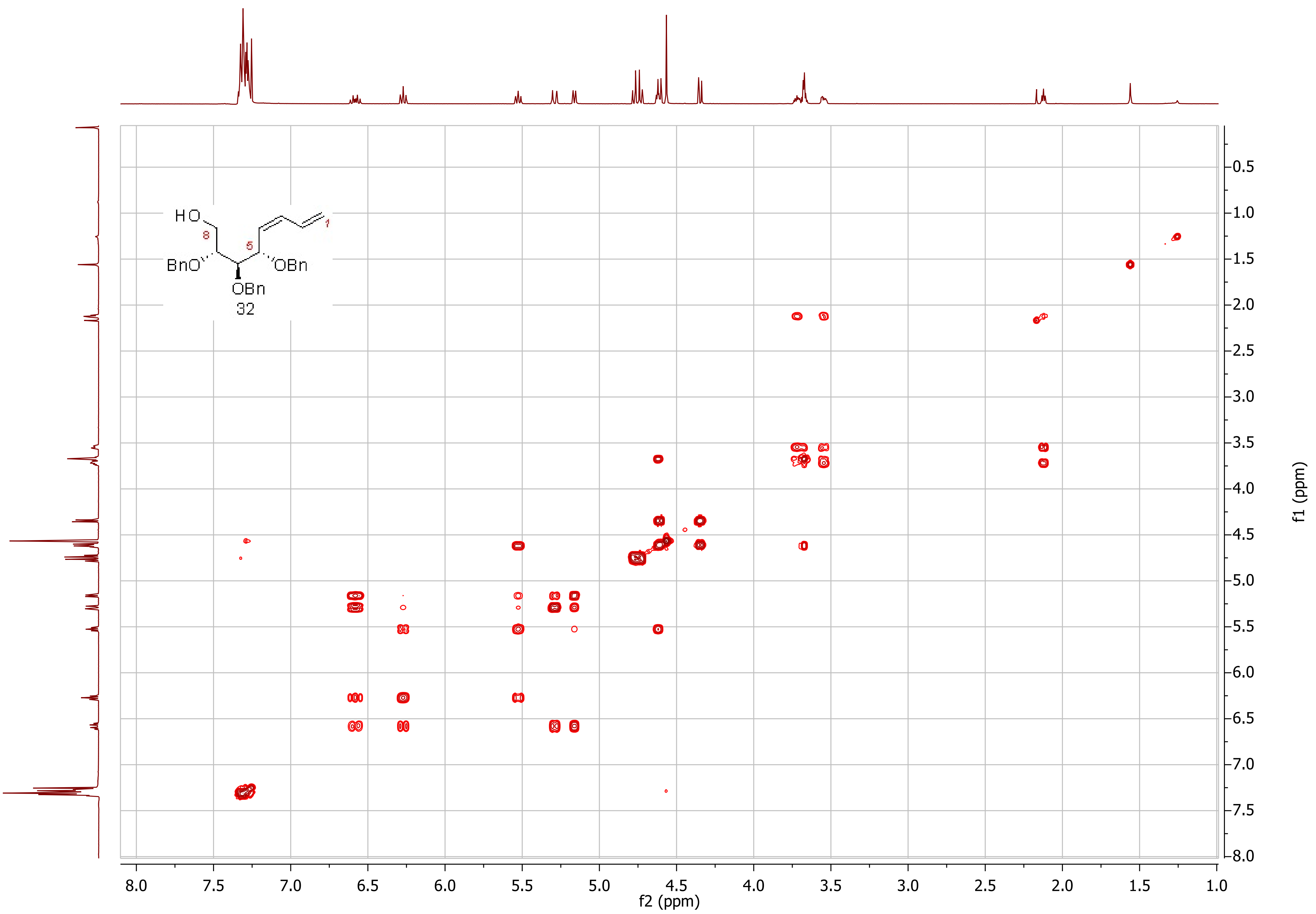
HO
8
5
1
BnO
OBn
OBn
32
f2 (ppm)
f1 (ppm)
8.0
7.5
7.0
6.5
6.0
5.5
5.0
4.5
4.0
3.5
3.0
2.5
2.0
1.5
1.0
0.5

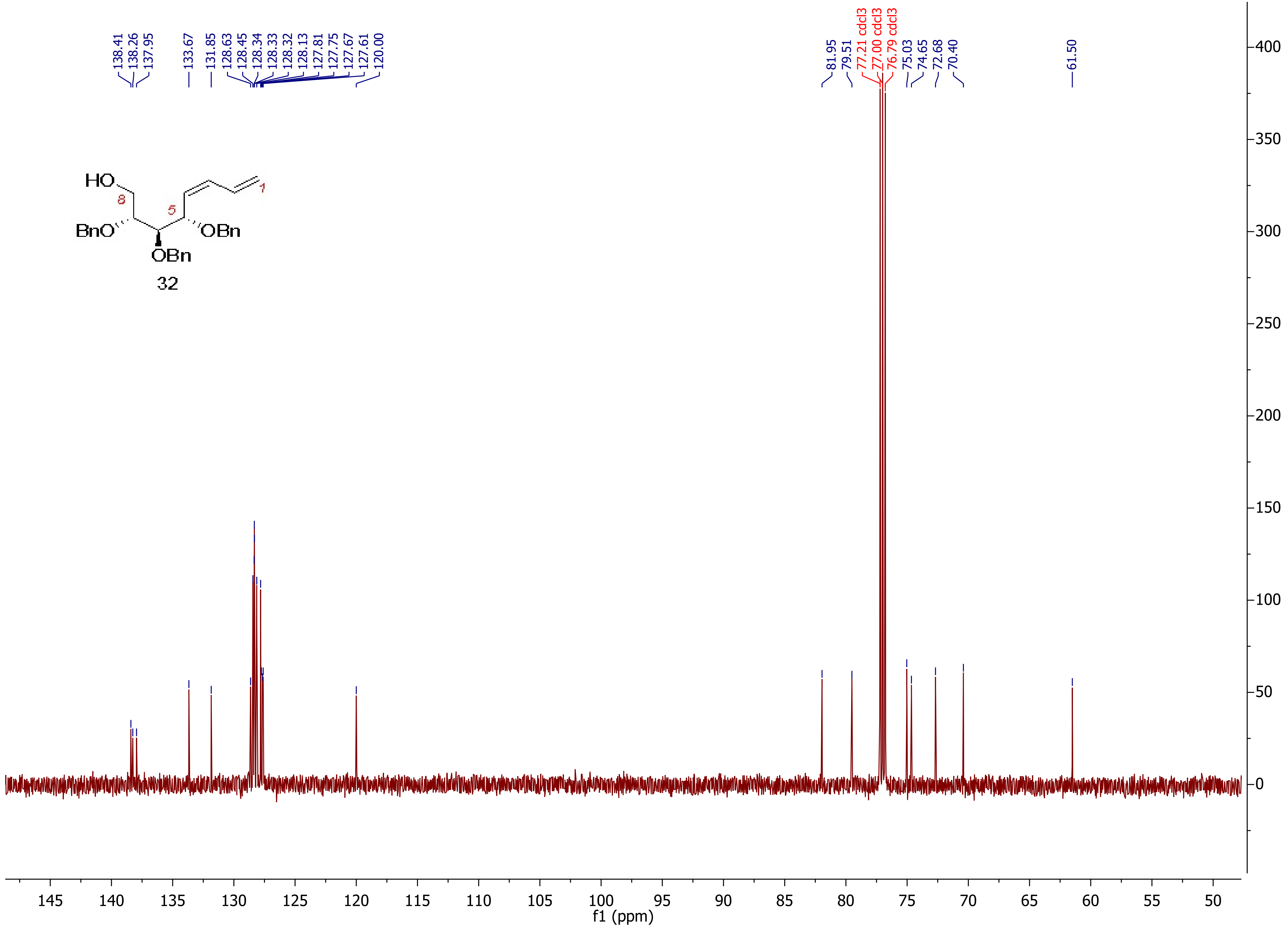
138.41
138.26
137.95
133.67
131.85
128.63
128.45
128.34
128.33
128.32
128.13
127.81
127.75
127.67
127.61
120.00
81.95
79.51
77.21 cdcl3
77.00 cdcl3
76.79 cdcl3
75.03
74.65
72.68
70.40
61.50
HO
8
5
1
BnO
OBn
OBn
32
f1 (ppm)

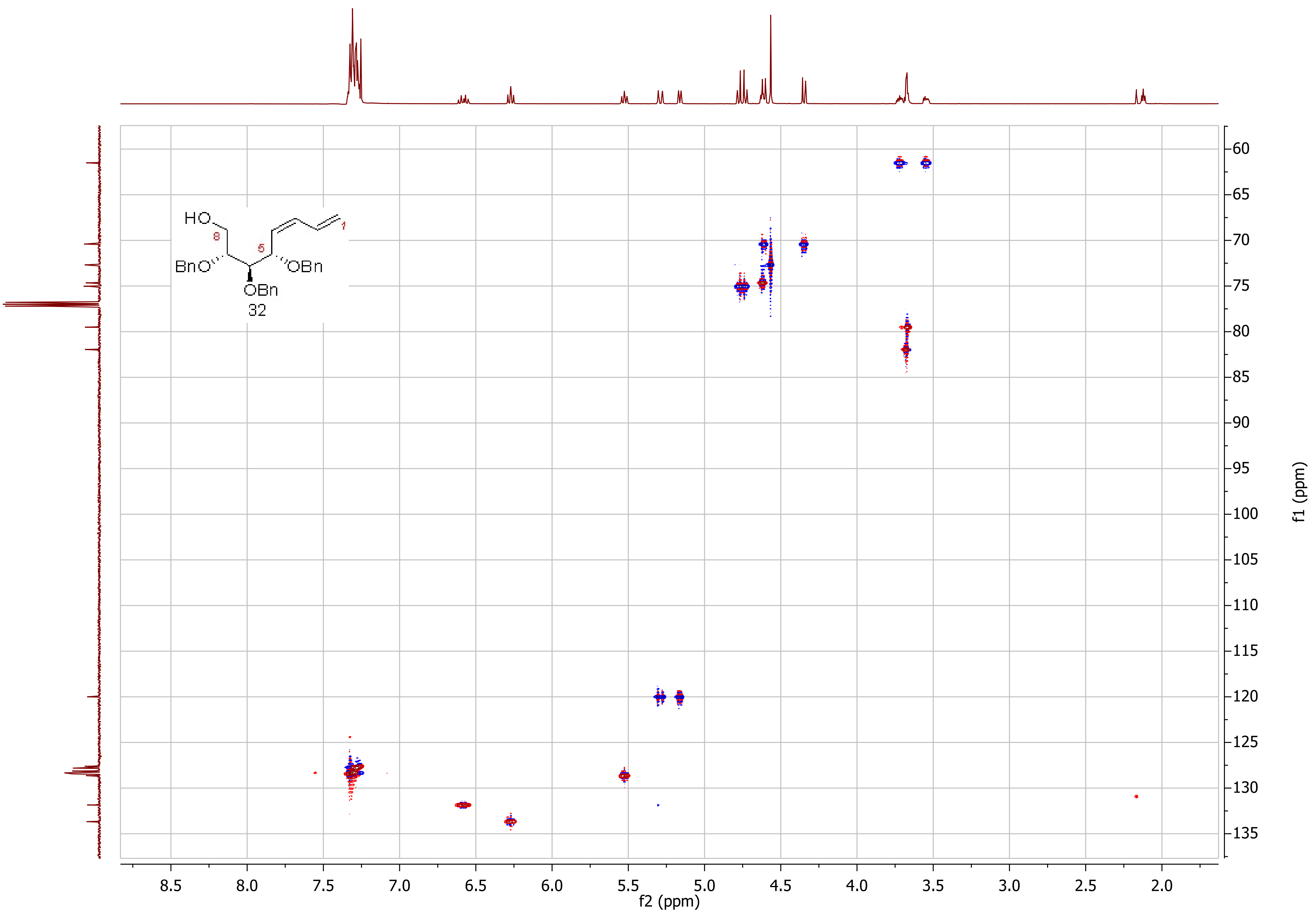

HO
8
5
1
BnO
OBn
OBn
32
f2 (ppm)
f1 (ppm)

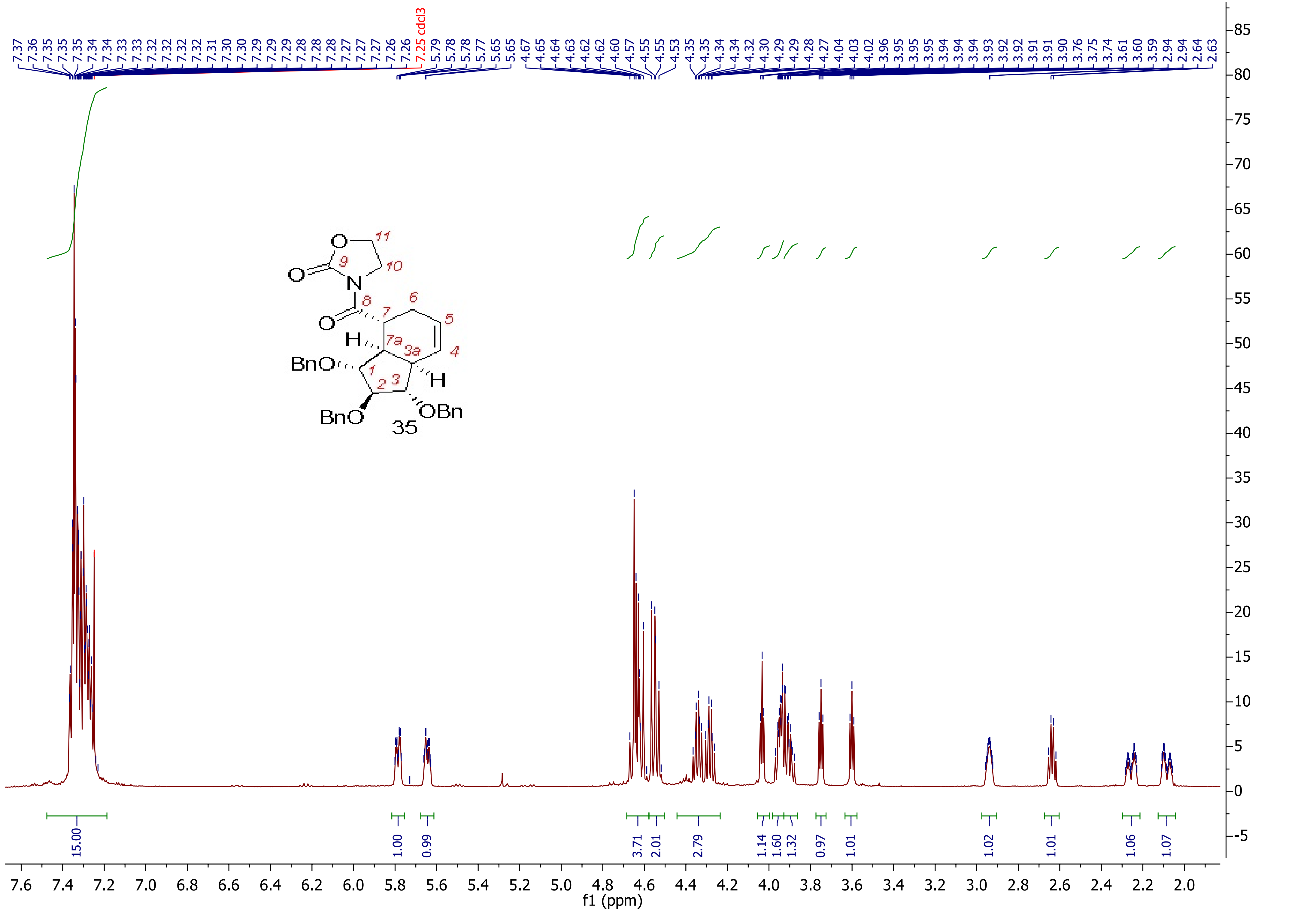
7.37 7.36 7.35 7.35 7.35 7.34 7.34 7.33 7.33 7.32 7.32 7.32 7.32 7.31 7.30 7.30 7.29 7.29 7.29 7.28 7.28 7.28 7.27 7.27 7.27 7.26 7.26
7.25 cdcl3
5.79 5.78 5.78 5.77 5.65 5.65
4.67 4.65 4.64 4.63 4.62 4.62 4.60 4.57 4.55 4.55 4.53 4.35 4.35 4.34 4.34 4.32 4.30 4.29 4.29 4.28 4.27 4.04 4.03 4.02
3.96 3.95 3.95 3.95 3.94 3.94 3.93 3.92 3.92 3.91 3.91 3.90 3.76 3.75 3.74 3.61 3.60 3.59 2.94 2.94 2.64 2.63
BnO
BnO
OBn
35
15.00 1.00 0.99 3.71 2.01 2.79 1.14 1.60 1.32 0.97 1.01 1.02 1.01 1.06 1.07
f1 (ppm)

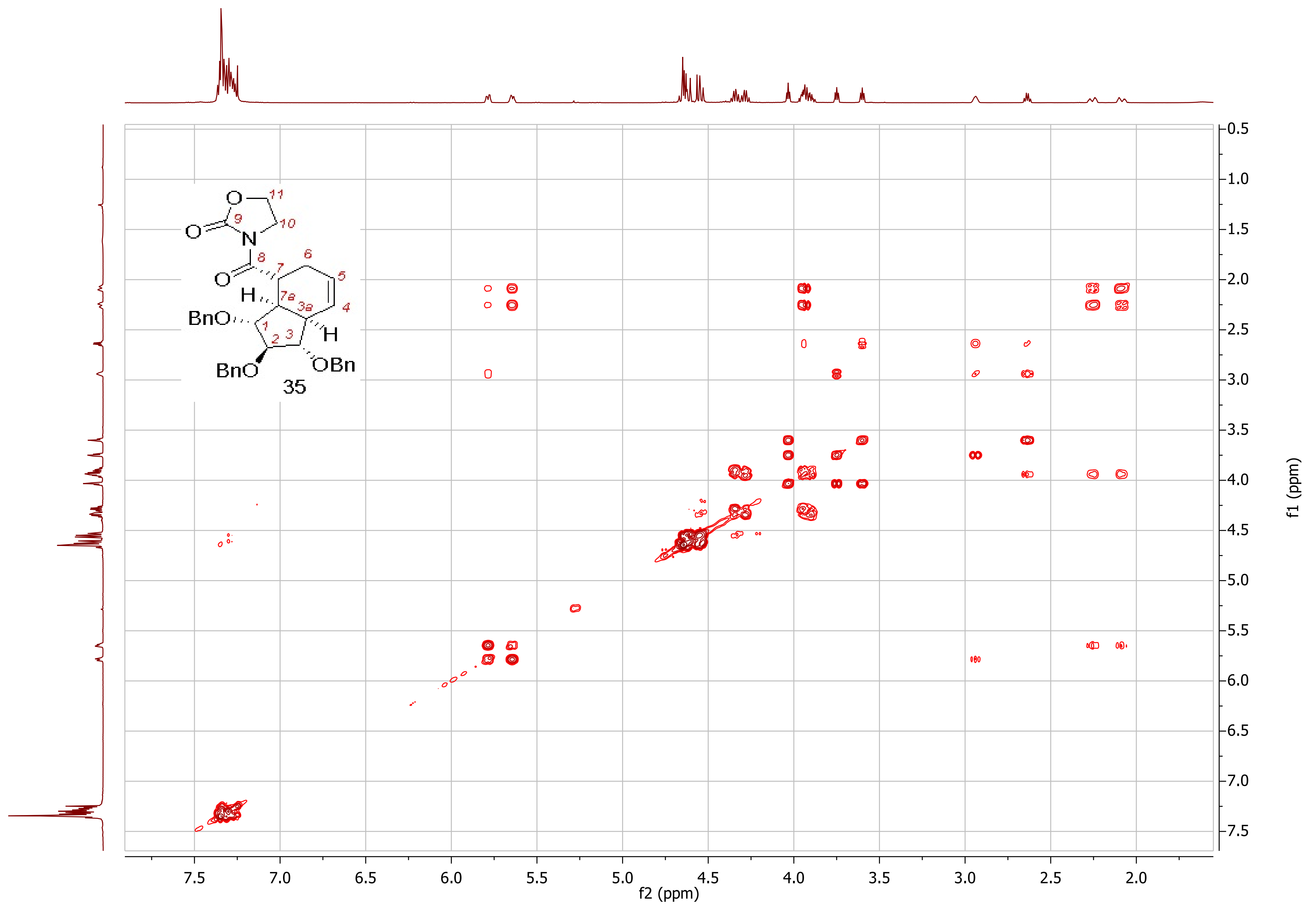

O
11
9
10
O
N
8
6
7
5
O
H
7a
3a
4
BnO
1
H
2
3
BnO
OBn
35
7.5
7.0
6.5
6.0
5.5
5.0
4.5
4.0
3.5
3.0
2.5
2.0
f2 (ppm)
0.5
1.0
1.5
2.0
2.5
3.0
3.5
4.0
4.5
5.0
5.5
6.0
6.5
7.0
7.5
f1 (ppm)

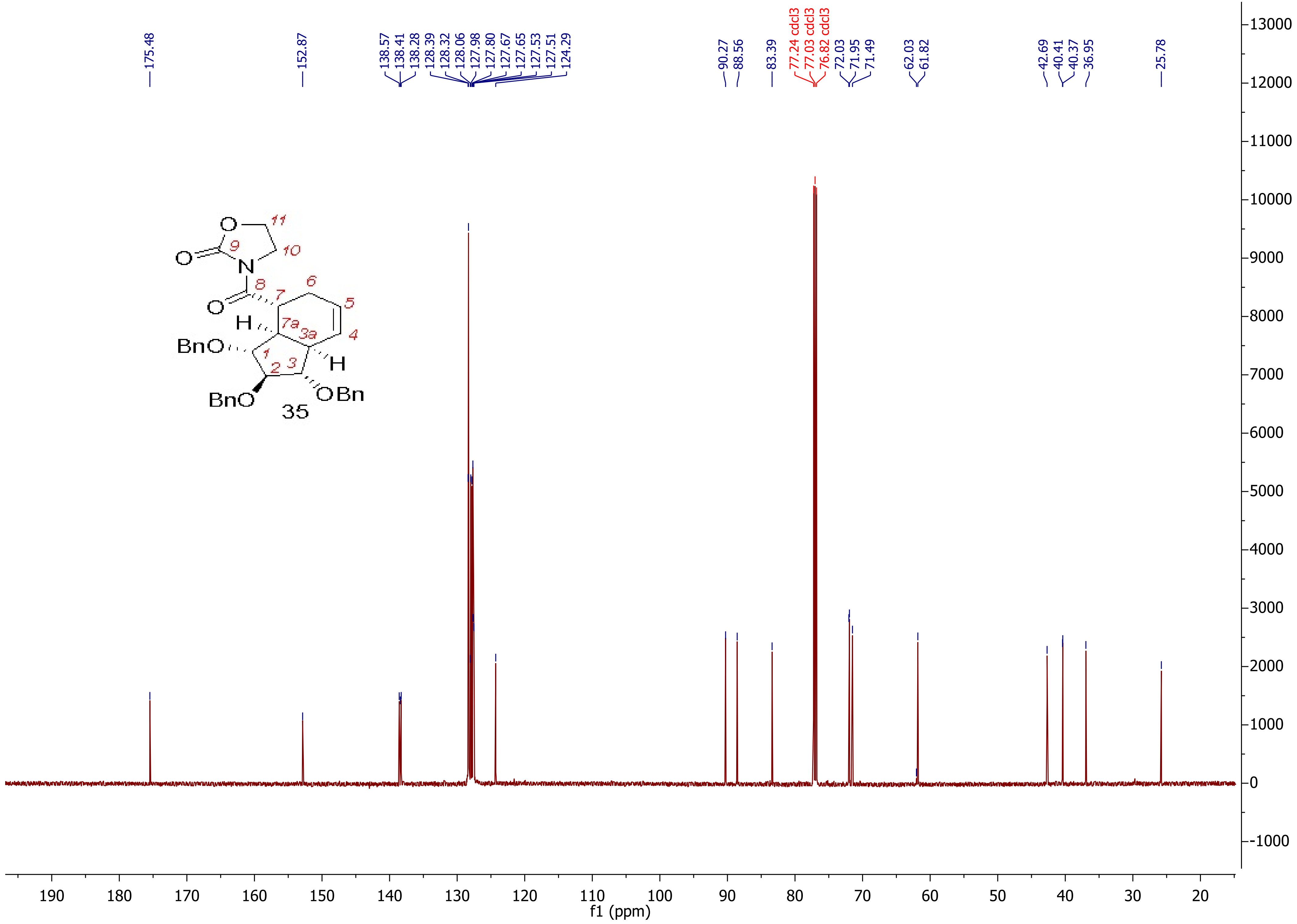
175.48
152.87
138.57
138.41
138.28
128.39
128.32
128.06
127.98
127.80
127.67
127.65
127.53
127.51
124.29
90.27
88.56
83.39
77.24 cdcl3
77.03 cdcl3
76.82 cdcl3
72.03
71.95
71.49
62.03
61.82
42.69
40.41
40.37
36.95
25.78
O
O
N
O
11
10
9
8
7
6
5
4
7a
3a
H
H
BnO
BnO
OBn
1
2
3
35
13000
12000
11000
10000
9000
8000
7000
6000
5000
4000
3000
2000
1000
0
-1000
190
180
170
160
150
140
130
120
110
100
90
80
70
60
50
40
30
20
f1 (ppm)

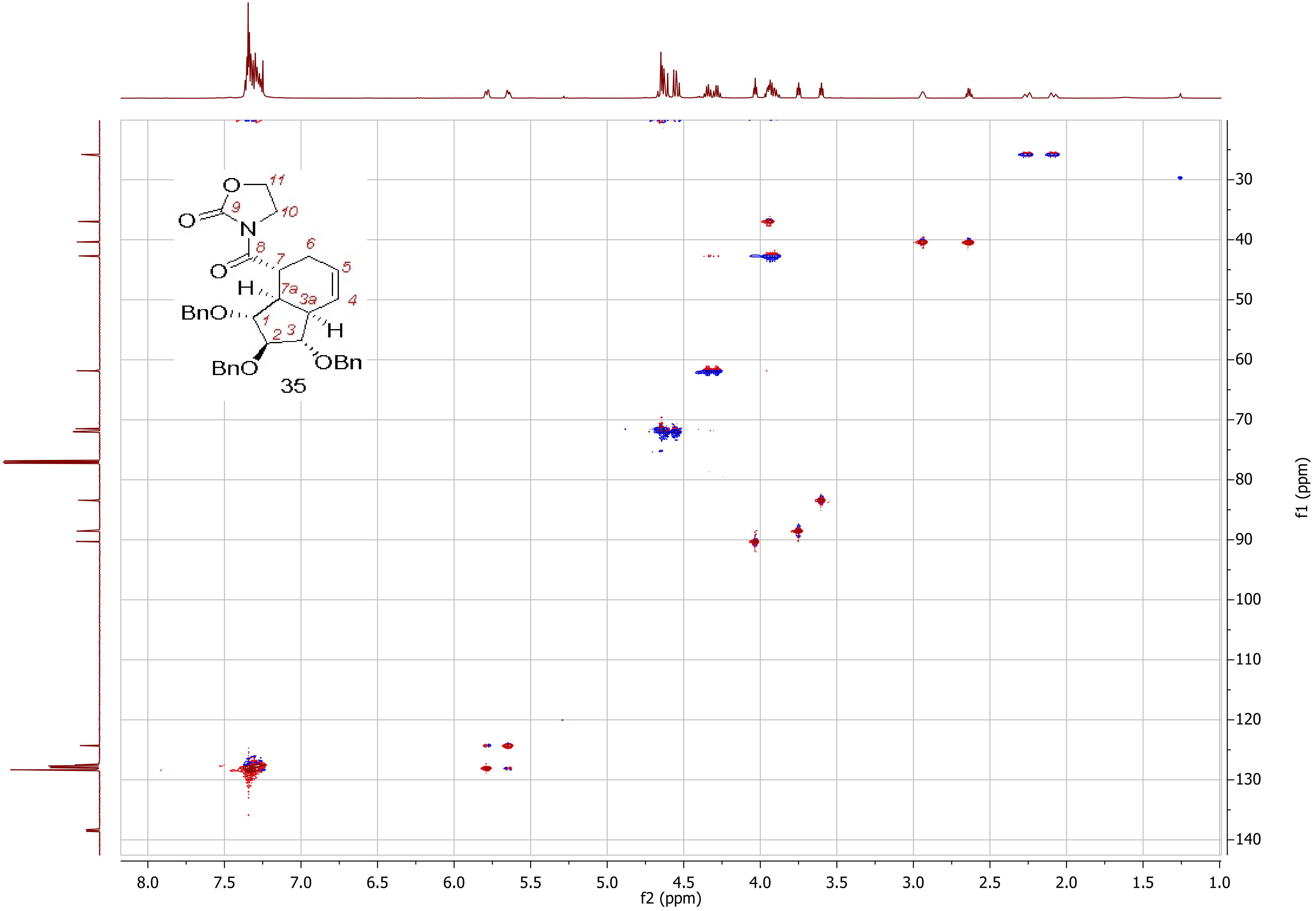

O
11
9
10
O
N
8
6
7
5
O
H
7a
3a
4
BnO
1
H
2
3
BnO
OBn
35
8.0
7.5
7.0
6.5
6.0
5.5
5.0
4.5
4.0
3.5
3.0
2.5
2.0
1.5
1.0
f2 (ppm)
30
40
50
60
70
80
90
100
110
120
130
140
f1 (ppm)
